# Potentially Toxic Element Migration Characteristics and Bioavailability in Soils of the Black Shale Region, Western Zhejiang Province, China

**DOI:** 10.3390/toxics13080679

**Published:** 2025-08-14

**Authors:** Huanyuan Chen, Baoliang Chen, Chunlei Huang, Xinzhe Lu, Ruosong Zou, Yutong Wei

**Affiliations:** 1 College of Environmental & Resource Sciences, Zhejiang University, Hangzhou 310027, China; 2 Qiantang Institute of Geology, Hangzhou 310027, China; 3 Zhejiang Nuclear Industry 262 Geological Brigade, Huzhou 313000, China; 4 Zhejiang Institute of Geosciences, Hangzhou 310007, China

**Keywords:** black shale, potentially toxic element migration, Cd bioavailability

## Abstract

Some soil heavy metal pollution, such as As (Arsenic) and Cd (cadmium), in the black shale areas of western Zhejiang, exhibits significant geological background characteristics, yet the migration patterns and bioavailability are unclear. This study systematically integrated geochemical investigations of the rock-weathered soil–water–soil system to reveal the migration mechanisms and the species of the potentially toxic elements (PTEs) in black shale regions. The results showed that strongly acidic drainage (pH = 3.9) released from black shale weathering led to significant enrichment of Cd and As in soils. The mean Cd concentration (0.84 mg/kg) was 3.3 times higher than the Zhejiang background value, with active speciation (exchangeable fraction and humic acid-bound fraction) dominating during migration. This research provides a scientific basis for PTE prevention and control in geologically high-background regions.

## 1. Introduction

Soil, as the core carrier for sustaining terrestrial ecosystem functions and ensuring food security, directly impacts human health and sustainable development [1]. In recent years, global attention has increasingly focused on natural heavy metal enrichment in soils triggered by geological backgrounds, with black shale series emerging as a key research target due to their unique elemental enrichment characteristics [2]. These organic-rich sedimentary formations, originating in reducing environments, are often associated with the anomalous enrichment of PTEs such as As, Cd, and Cu (Copper) [3,4]. During supergene weathering processes, black shale series release PTEs through cascade reactions involving sulfide oxidation, organic matter decomposition, and silicate mineral alteration [5], leading to the significant elevation of regional soil heavy metal baseline values and posing risks to agricultural product safety via crop uptake [6]. In the study area of western Zhejiang, rice is the dominant staple crop, and PTEs in soil can migrate through the soil–rice system, accumulating in edible parts and ultimately threatening human health via the food chain. Therefore, investigating the migration and bioavailability of PTEs in both soil and rice is critical to comprehensively assess the ecological and health risks of geogenic PTE enrichment. In southern China, the extensively distributed Cambrian black shale regions have become focal areas for PTE pollution control, with their environmental effects holding critical scientific and practical significance [2].

PTEs accumulation in soils not only directly threatens crop safety but also disrupts critical biogeochemical cycles and soil biological communities. For instance, long-term exposure to heavy metals can alter the diversity and activity of nitrogen-cycling microorganisms, with denitrifying bacteria showing adaptive enrichment under high contamination levels, potentially accelerating nitrite reduction and disrupting the balance of nitrogen transformation [7]. Meanwhile, the soil biota exhibits altered community structures under PTE stress: changes in the maturity index, genera richness, and feeding group abundance reflect disturbances, even in regions where PTE effects are overshadowed by other soil properties like pH and moisture [8]. These cascading effects highlight the need to understand not only heavy metal migration but also their ecological impacts on soil function.

The PTE migration driven by black shale weathering involves multi-interface coupled processes. During rock weathering, acidic media generated by pyrite oxidation not only accelerate mineral decomposition but also significantly alter the speciation of PTEs by modulating soil pH [9]. Studies in areas like Chung-Joo, South Korea, have revealed a strong positive correlation between PTE concentrations in black shale-derived soils and parent rocks; however, the evolution of this correlation during supergene geochemical processes remains unclear [10]. Additionally, investigations in China’s Yangtze River Delta region indicate that deep soil PTE distribution aligns closely with geological backgrounds, suggesting potential contributions of natural sources to surface pollution [2]. Nevertheless, existing research predominantly focuses on single-medium analyses, lacking systematic insights into elemental migration fluxes, speciation transformation mechanisms, and bioavailability regulation within the rock–soil–crop continuum.

This study, based on analyzing the PTE migration characteristics in the rock-weathered soil–water–soil system of a typical black shale-developed region, addresses core scientific questions: the speciation transformation pathways and dominant controlling factors of PTEs during black shale weathering processes. By integrating analyses of soil and rice samples, this study aims to clarify the complete migration chain of PTEs from black shale to soil and then to crops, providing a more holistic understanding of geogenic pollution risks. The results provide theoretical support for formulating source reduction strategies and farmland remediation protocols.

## 2. Materials and Methods

### 2.1. Study Area Overview

Zhejiang Province, as a pioneer in China in conducting multi-scale geochemical surveys and agricultural land pollution remediation trials, has implemented a series of soil environmental investigations and monitoring programs over the past two decades. Notably, during 2015–2019, the Geochemical Monitoring of Cultivated Land Environmental Quality Evolution in Basic Farmland was carried out (see Supplementary Material Text S1). These efforts have accumulated critical datasets on PTE contamination, soil acidification, and trace element dynamics, providing foundational data for this study and practical remediation efforts.

The study area is in the tectonic intersection zone between the Yangtze Para platform and the South China Fold Belt (Supplementary Material Figure S1). Its geological evolution has been controlled by the Jiangshan-Shaoxing Deep Fault, resulting in significant stratigraphic differentiation between northwestern Zhejiang (Jiangnan Stratigraphic Region) and southeastern Zhejiang (South China Stratigraphic Region) [11,12]. The primary soil parent materials consist of Cambrian black shale formations (containing stone coal and polymetallic mineralization) and Yanshanian acidic magmatic rocks. A series of 1:250,000-scale geochemical surveys reveal distinct elemental associations between plain areas (marine parent materials) and mountainous–hilly areas (Paleozoic sedimentary/volcanic parent materials): MgO-CaO-Cl (Chlorine) and Mo (Molybdenum)-Se (Selenium)-As. These differences are governed by geochemical inheritance from parent rocks (e.g., marine sedimentation enriching base elements), human farming activities (N (Nitrogen)-P (Phosphorus) correlations with organic matter), and supergene geochemical processes (Cr (Chromium)-Hg (Mercury) pollution migration) [13]. The complex geological background and geomorphic patterns (Supplementary Material Figures S2 and S3) collectively shape a red-yellow paddy soil composite distribution system. Detailed stratigraphic sequences, magmatic activity phases, and elemental spatial differentiation mechanisms are provided in Supplementary Material Text S2.

### 2.2. Field Investigation and Sampling

#### 2.2.1. Geochemical Survey and Soil Sampling

**Figure 1:**
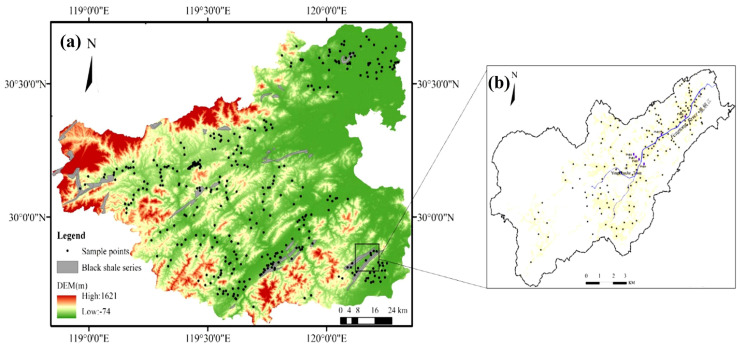
A 1:50,000−scale sampling location map of surface soil samples (0–20 cm) in different black shale series development areas (**a**); Including Zhuji area (**b**).

The soil geochemical survey and topsoil sampling followed the Technical Specification for Geochemical Evaluation of Land Quality (Ministry of Land and Resources P.R.C/Specification of land quality geochemical assessment/DZ/T 0295—2016/2016). Surface soil samples (0–20 cm) were systematically collected from black shale-developed areas in Zhuji city, Changshan county, and other regions, with sampling locations illustrated in Figure 1. Sampling details are described in Supplementary Material Text S2.

#### 2.2.2. Typical Soil Profile Sampling

To investigate the physicochemical properties of cultivated soils (e.g., soil type, parent material, texture, and pH), rock-weathered soil–soil profile investigations were conducted in the Changshan and Zhuji regions. Three black shale profiles (CS003, CS009, and CS0010, a total of seven profiles and 29 subsamples) were selected for analysis. The sampling details are provided in Supplementary Material Figure S2 and Table S1, Text S3, and Figure S3. The weathering profiles were stratified as follows: Layer A (topsoil layer), Layer B (highly weathered layer), and Layer C (moderately weathered layer). Detailed descriptions of each layer are in Supplementary Material Text S3 and Figure S4.

#### 2.2.3. Water Sample Collection

In typical black shale areas, 8 samples of surface water (near exposed black shale), farmland irrigation water, Changshan River water, and rainwater were systematically collected and categorized. Sampling locations and basic information for water samples in the Changshan black shale exposure area are provided in Supplementary Material Figure S5 and Table S2.

#### 2.2.4. Rice Sample Collection

Rice plant components were rinsed with distilled water onsite, oven-dried in the laboratory, and weighed to determine dry mass. Theoretical rice yields were calculated and used for subsequent experimental analysis.

### 2.3. Sample Analysis

All analytical testing was conducted at the Hangzhou Mineral Resources Monitoring Center (Ministry of Natural Resources). Strict quality control protocols were implemented, and analytical quality reports were issued for all samples.

#### 2.3.1. Total Element and Physicochemical Analysis of Soils and Crops

Soil and crop samples were pretreated and analyzed as follows:

(1) Cd, Cu, Ni (Nickel), and Se: Inductively coupled plasma mass spectrometry (ICP-MS);
(2) Pb, Zn: X-ray fluorescence spectrometry (XRF);
(3) As: Hydride generation atomic fluorescence spectrometry (HG-AFS);
(4) Hg: Cold vapor atomic fluorescence spectrometry (CV-AFS);
(5) pH: Glass electrode method;
(6) Organic matter: High-frequency heating infrared absorption method.

Detailed procedures are provided in Supplementary Material Text S4.

#### 2.3.2. Water Sample Analysis

Irrigation water was analyzed for 11 parameters: pH, sulfate, nitrate, nitrite, Zn, Cu, Cr (VI), Pb, Hg, As, Cd, and Cations (Na^+^, Ca^2+^, K^+^, etc.) and Anions (Cl^−^, F^−^, SO_4_^2−^, HCO_3_^−^, etc.) following the Technical Requirements for Eco-geochemical Sample Analysis (DD2005-03) and supplementary guidelines. Analytical methods are listed in Supplementary Material Table S3.

#### 2.3.3. Extraction Methods of Soil and Crop Components

For the sequential extraction of different forms in soil (and crops, if applicable), the following seven-step extraction procedure was adopted:

1. Water-soluble form

Extractant and operation steps: Use 60 mL of distilled water with pH = 7. Continuously shake at 30 °C for 2 h, then equilibrate for 2 h.

2. Exchangeable form

Extractant and operation steps: Take 60 mL of 1 mol/L NH_4_Ac solution. Continuously shake at 30°C for 2 h and then equilibrate for 2 h.

3. Carbonate-bound form

Extractant and operation steps: Use 60 mL of 0.1 mol/L EDTA (pH = 6.5). Perform intermittent shaking at 30 °C for 2 h, followed by a 2 h equilibration.

4. Weakly crystalline Fe-Mn oxide-bound form

Extractant and operation steps: First, use 60 mL of 0.1 mol/L NH_2_OH·HCl. Continuously shake at 30 °C for 2 h. Then, use 60 mL of 0.1 mol/L NH_4_Ac. Equilibrate at 30 °C for 2 h.

5. Crystalline Fe-oxide form

Extractant and operation steps: Take 60 mL of 2 mol/L HCl. Continuously shake at 30 °C for 2 h and then equilibrate for 2 h.

6. Organic-bound form

Extractant and operation steps: Use 60 mL of 10% H_2_O_2_-2 mol/L HCl. Shake at 30 °C for 2 h and then equilibrate for 2 h.

7. Residual form

Treatment: Conduct digestion with HF-HNO_3_-H_2_SO_4_.

8. Experimental Operation

Weigh 5 g of air-dried natural soil and crop (particle size < 0.180 mm) and fully mix it with 1 g of dry PTE in a 100 mL polyethylene vial. Then, add 0, 0.1, 0.2, 0.3, 0.4, and 0.5 g of activated carbon in sequence, and add 5 mL of distilled water. Let it stand stably for 30 days. According to the above extraction steps, add the extractants for each step, then shake on a water bath constant temperature shaker. After that, filter, fully wash the residues of each step, and analyze and determine the PTE content in the filtrate. For each group of experiments, 3 parallels were set simultaneously, and the reported result is the average value of the 3 parallel samples [14,15].

### 2.4. Analytical Quality Control

#### 2.4.1. Soil Sample Quality Control

Duplicate samples were blindly selected at a rate of 6.0% for repeatability testing. The relative double difference (RD% = 2 × |A − B|/(A + B) × 100%) was calculated between the original analytical data (A) and duplicate test data (B). Per the standard, RD% ≤ 30% was deemed acceptable for original data within threefold of the detection limit (DL), while RD% ≤ 25% was required for concentrations exceeding threefold of the DL. For pH, an absolute deviation (|A − (A + B)/2|) < 0.1 was considered acceptable. Statistical results showed that the pass rates for all elements exceeded 90%, complying with the requirements of the Ministry of Land and Resources P.R.C/Specification of multi-purpose regional geochemical survey (1∶250000)/DZ/T 0258-2014/2014.

Additionally, 5% of samples were selected for external validation, with all tested indicators achieving pass rates exceeding 93%.

#### 2.4.2. Crop Sample Quality Control

Two national Grade I reference materials—GBW10043 (GSB-21 Liaoning rice) and GBW10044 (GSB-22 Sichuan rice)—were selected. Each sample was analyzed 12 times, and the relative error (RE%) between the mean measured values and certified reference values was calculated, with RE% ≤ 20% set as the accuracy criterion. Additionally, two similar reference materials (GSB-6 and GSB-11) were repeatedly analyzed 12 times to calculate the relative standard deviation (RSD) for each element, with RSD ≤ 15% defined as the precision criterion. Results demonstrated that both the precision and accuracy of the analytical method met regulatory requirements.

### 2.5. Data Processing Methods

SPSS 19.0, Excel 2010, and Origin 9.0 were used for data processing and analysis, while Arcgis 10.0 and Mapgis 6.7 were employed for graphical processing.

### 2.6. Technical Standards and Regulations

The sample collection, experimental analysis, and quality assessment strictly adhered to Chinese national/industry standards (Supplementary Material Text S5) to ensure normative data acquisition and interpretation.

## 3. Results and Discussion

### 3.1. Distribution and Accumulation Characteristics of PTEs in Typical Black Shale Soils

Based on 1:50,000-scale geochemical data from Zhejiang Province, 5172 soil samples derived from Cambrian Hetang and Dachenling Formation black shales were analyzed (Table 1). The mean concentrations of PTEs were: As (24.47 mg/kg), Cd (0.84 mg/kg), Cr (78.20 mg/kg), Cu (39.25 mg/kg), Hg (0.14 mg/kg), Ni (37.60 mg/kg), Pb (45.77 mg/kg), and Zn (129.01 mg/kg). As, Cd, and Ni levels significantly exceeded Zhejiang’s background values (by 257%, 236%, and 20%, respectively), while Cr and Zn slightly exceeded background values (by 1.6% and 3.3%, respectively). In contrast, Hg, Cu, and Pb were lower than background values (by 82.5%, 21.0%, and 21.2%, respectively) [16]. Studies indicate that Cd and As enrichment in black shale soils likely originates from parent rock weathering [17,18].

A Pearson correlation analysis (Supplementary Material Table S4) revealed that Cd exhibited a certain correlation with Zn, Pb, and Mn, (r > 0.4, *p* < 0.01). Liu, et al. [19] suggested that this may be controlled by coprecipitation with sulfide minerals (e.g., sphalerite, galena) or adsorption migration during supergene oxidation processes. In contrast, the certain correlation between As and Cu/Ni (r > 0.3, *p* < 0.01) might relate to the synchronous fixation of Cu^2+^ and Ni^2+^ under the coordination of As (V) with α-FeOOH surface hydroxyl groups, achieving an adsorption capacity of 18.7 mg/g [20]. Notably, the high correlation between Zn and Pb (r = 0.726, *p* < 0.01) may indicate superimposed anthropogenic inputs (e.g., e-waste dismantling activities) [21], though further validation with complementary tracing methods is required.

**Figure 2:**
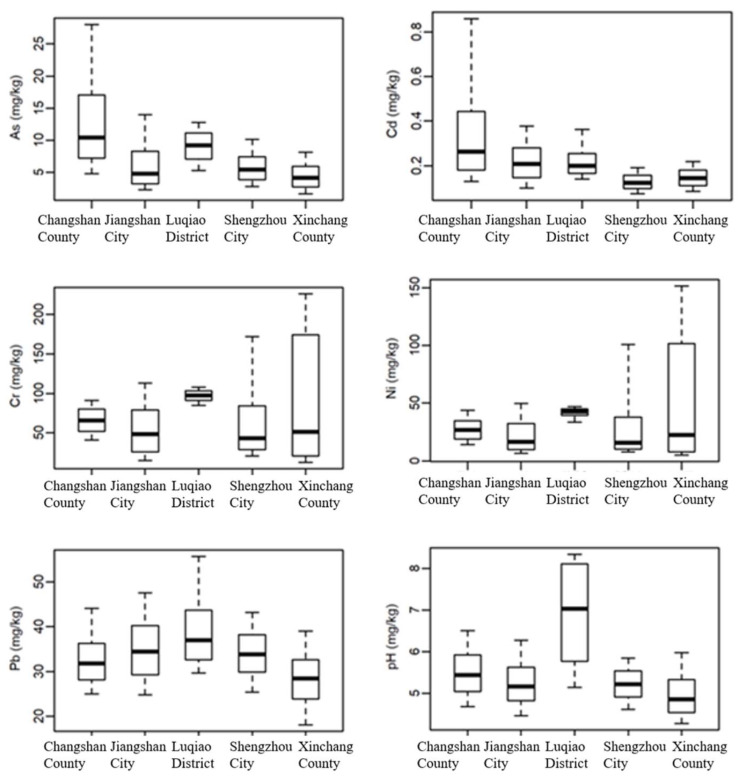
Comparison of PTE contents across regions (box plot whiskers represent the 1/10 and 9/10 percentiles; upper and lower box edges denote the 1/4 and 3/4 quartiles; and the bold line within the box indicates the median).

To compare PTE accumulation characteristics across different regions, box plots were generated for Changshan–Jiangshan (black shale area), Luqiao (anthropogenically polluted area), and Shengzhou–Xinchang (basalt area) (Figure 2). The study found that Cd and As were the most prominent high-accumulation PTEs in black shale regions, with Changshan–Jiangshan soils exhibiting the highest median concentrations (1.2 mg·kg^−1^ for Cd and 32.5 mg·kg^−1^ for As), demonstrating a significant regional representativeness (Figure 2) consistent with prior findings [22]. In Shengzhou–Xinchang soils, Cr and Ni were most elevated, likely linked to local industrial activities and the high geological background of basalt. Meanwhile, Luqiao soils exhibited the highest Pb levels, along with elevated Cr, Ni, and As, indicating substantial anthropogenic pollution impacts from e-waste dismantling [23].

### 3.2. Mineral Composition and PTE Migration/Accumulation Characteristics in Typical Black Shale Weathering Profiles

To investigate the mineral composition and PTE distribution/accumulation patterns in weathering profiles of the Cambrian Hetang Formation black shale in Changshan, we analyzed the major and trace element contents across layers of profiles CS003, CS009, and CS0010 (Supplementary Material Tables S5-1 and S5-2).

Based on variations in the Chemical Index of Alteration (CIA), Residual Coefficient (Rc), and metal oxide contents (CaO, MgO, Na_2_O, etc.), we observed differing weathering intensities across profile layers: CS0010 > CS009 > CS003. The differentiation of major elements between layers may relate to kinetic processes involving preferential carbonate mineral dissolution [24,25] (see Supplementary Material Text S6 for detailed mineral migration and weathering characteristics).

As shown in Tables S5-2 and S6, trace elements in the western Zhejiang black shale weathering profiles exhibit significant differentiation, likely driven by redox-induced water–rock interactions after black shale exposure (mechanism illustrated in Supplementary Material Figure S6 and Text S7). Overall, the chemical weathering of black shale displays cyclic progressive characteristics, marked by leaching of Ni, Cu, Zn, Cd, and Se and enrichment of TOC, Cr, and Hg.

**Figure 3:**
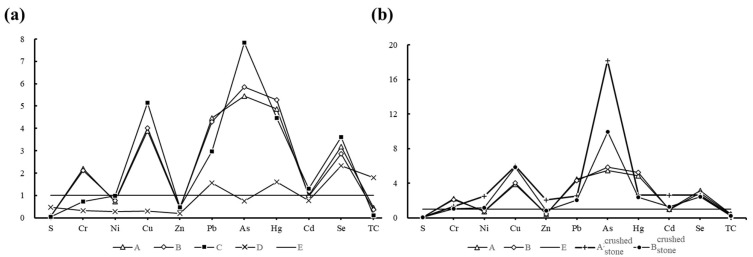
Variation curves of trace elements in soil relative to bedrock concentrations in the CS003 weathering profile (**a**); variation curves of trace elements in rock fragments and corresponding soil layers relative to bedrock concentrations (**b**). The meanings represented by each capital letter: A (Eluviation Zone Topsoil Layer), B (Eluviation Zone Highly Weathered Layer), C (Illuviation Zone Moderately Weathered Layer), D (Transition Zone Weakly Weathered Layer), and E (Parent Rock Zone Fresh Bedrock Layer).

To systematically investigate elemental migration characteristics in the weathering profiles of western Zhejiang black shale, we adopted the methodology of Bruemmer, et al. [26] and classified the profiles into five zones—A (Eluviation Zone Topsoil Layer), B (Eluviation Zone Highly Weathered Layer), C (Illuviation Zone Moderately Weathered Layer), D (Transition Zone Weakly Weathered Layer), and E (Parent Rock Zone Fresh Bedrock Layer)—based on idealized element distribution patterns. Elements were categorized as leached, illuviated, or residual according to their geochemical behaviors ([27]; see Supplementary Material Text S7). For the CS003 profile (Figure 3a), As exhibited maximum enrichment in Layer C (7.83× parent rock levels) and minimum in Layer A (5.45×), while S was significantly leached. Ni, Cu, Zn, As, Cd, and Se displayed illuviation traits, whereas Cr and Pb persisted as residual elements, with overall elemental deviations from parent rock concentrations averaging 3.37×. Further analysis (Figure 3b) revealed contrasting microelement ratios between parent rock fragments and soils in Layers A and B: Group I (Cr, Pb, Hg, and Se) showed higher soil concentrations, while Group II (Ni, Cu, Zn, As, and Cd) exhibited the inverse. Notably, despite being illuviated, Group II elements had far higher enrichment rates in Layer A than in Layer C.

**Figure 4:**
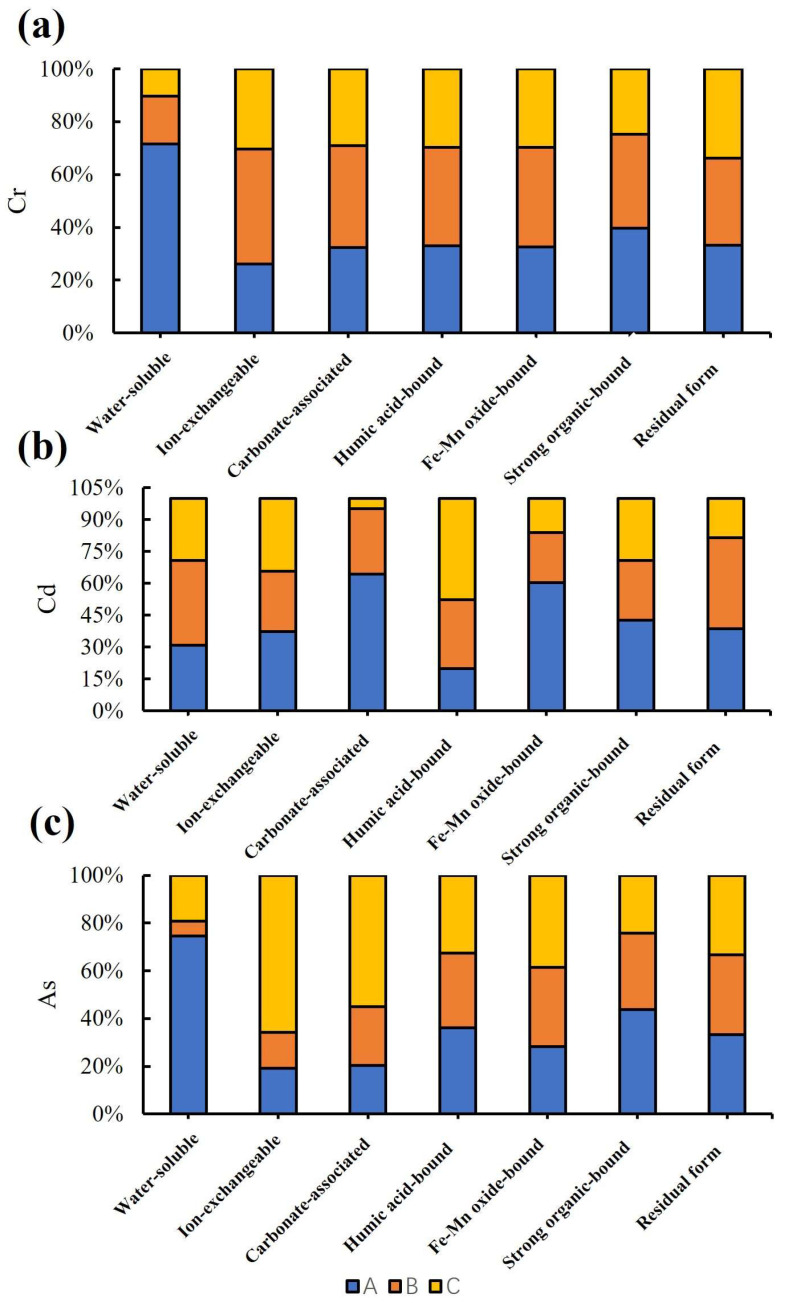
Relative contents of seven-step sequential extraction fractions for Cr (**a**), Cd (**b**), and As (**c**) across layers in the CS010 profile.

To elucidate the migration states of PTEs in the eluviated layers (A, B) and moderately weathered layer (C) of black shale weathering profiles in western Zhejiang, soil samples from Layers A, B, and C of the CS010 profile were analyzed using a seven-step sequential extraction method to determine the speciation of Cr, Cd, and As (Supplementary Material Table S7). As shown in Figure 4, Cr and As were predominantly present in the residual fraction (≈ 90% of total content), with As primarily bound to humic acid and Fe-Mn oxides, while Cr showed strong associations with organic matter followed by Fe-Mn oxides. Cd mainly existed in ion-exchangeable, humic acid-bound, and residual fractions, with negligible contributions from other forms. As weathering intensity increased, the ion-exchangeable Cd fraction rose, the humic acid-bound fraction declined, and the residual fraction initially increased before slightly decreasing. Detailed discussions on speciation composition and migration mechanisms are provided in Supplementary Material Text S8.

In summary, the study revealed that elements such as Cr, Ni, Cu, Zn, Pb, As, Hg, Cd, and Se in black shale weathering profiles exhibit significant chemical activity, with their enrichment/depletion patterns effectively indicating the weathering intensity. A higher weathering intensity correlates with greater disparities in trace element concentrations between soil and parent rock fragments: Ni, Zn, and Cd preferentially remain in rock matrices, while Cr, Pb, Hg, and Se migrate dominantly into soils.

### 3.3. Migration Characteristics of PTEs in Surface Runoff and Irrigation Water

Based on extensive prior research and the findings of Section 3.2, exposed black shale rapidly generates strongly acidic wastewater with PTE leaching during weathering [28]. To track its environmental impacts, we systematically investigated water bodies (rainwater, mountain streams, mine wastewater, and river water) near Quaternary deposits in the Changshan River Basin black shale area.

#### 3.3.1. Elemental Characteristics of Surface Water

Data (Supplementary Material Table S8) revealed significant differences in the geochemical characteristics of various water bodies in black shale areas compared to the Farmland Irrigation Water Quality Standard (GB5084-2021): rainwater, mountain streams, and Changshan River water (unaffected by black shale) complied with irrigation standards, while mine wastewater from black shale-exposed areas exhibited low pH (3.9) and excessive Cd and Zn levels (Cd content exceeded the standard by 18.4 times). Hydrochemical comparisons showed that Changshan surface water was weakly alkaline (pH = 7.6–7.9), with river water characterized by higher ionic diversity (e.g., elevated Na^+^, Ca^2+^, K^+^, Cl^−^, F^−^, and SO_4_^2−^) compared to mountain streams, which were enriched with HCO_3_^−^, Fe, and Mg. Runoff in black shale areas displayed extreme geochemical anomalies—strong acidity (pH = 3.9), high sulfur content, and PTE enrichment aligned with the composition of elements leached during black shale weathering.

#### 3.3.2. PTE Characteristics in Agricultural Soils of Typical Black Shale Watersheds

**Figure 5:**
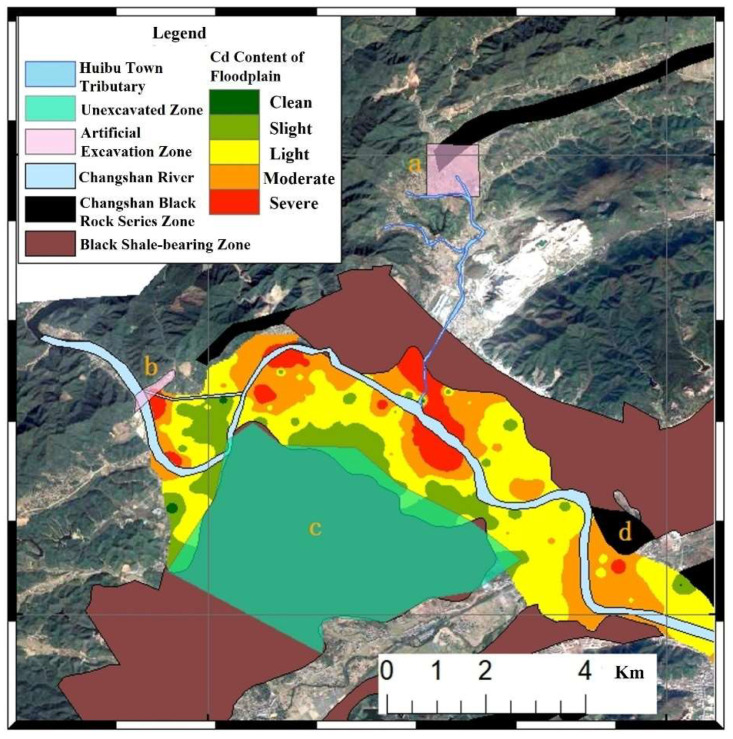
Distribution of local anthropogenic environments and Cd concentrations in Quaternary deposits of the Changshan River Basin black shale area.

The study area’s hydrology features 60+ tributaries, uneven rainfall runoff distribution (predominantly April–September), and flash flood dynamics. To assess riverine impacts on soil PTEs, inverse distance weighting (IDW) interpolation was applied to Cd concentrations in farmland soils near Quaternary deposits (Figure 5), identifying four high-enrichment zones (a, b, c, and d) (geological contexts in Supplementary Material Text S9):

Zone a: Northern enrichment linked to artificially excavated black shale areas, where acidic metal-rich solutions migrate along river channels; southern enrichment stems from suspended sediment deposition at tributary confluences (convex bank deposition effect).

Zone b: Southern enrichment from westward inflow of Zone a water depositing on eastern banks; northern enrichment corresponds to convex bank deposition on southern riverbanks.

Zone c: Enrichment driven by weathering wastewater inputs from Hetang Formation coal mines upstream of Huibu Town tributaries, causing widespread soil contamination.

Zone d: Likely caused by secondary pollution from exposed black shale due to construction activities in residential, mountainous, forested, and farmland areas. 

Analysis of elemental migration and distribution patterns revealed that variations in element concentrations in the Changshan River are primarily governed by weathering-derived solutions from the black shale series. Comprehensive studies delineate the region’s environmental geochemical characteristics as follows:

(1) The Changshan River water is weakly alkaline (pH 7.6–7.9) and is characterized by high levels of Na^+^, Ca^2+^, K^+^, Cl^−^, F^−^, and SO_4_^2−^. The mountain streams have high levels of HCO_3_^−^, Fe, and Mg; the mine wastewater in the exposed black rock series area, with its strong acidity, high PTE (Cd, As) content, and high SO_4_^2−^ levels, directly leads to a decrease in the pH of the surrounding soils, an increase in the total amount of Cd/As, and an increase in the proportion of exchangeable Cd. The mountain streams in non-exposed areas have a clean water quality, corresponding to low PTE content and stable speciation in the soil.
(2) The outcrop location of the black rock series controls the PTE pollution source. Surface runoff serves as the migration carrier, and its flow velocity and discharge determine the migration distance of PTEs in water and the scope of soil pollution. The speciation of PTEs in the solution (such as ionic state, particle-bound state) affects their occurrence forms in the soil (exchangeable state, residual state, etc.) [29].
(3) The river sedimentation process causes PTEs (such as Cd, Pb) carried by suspended particles in water to accumulate in the soil, resulting in a significantly higher total amount of PTEs in the soil of the deposition area than in the non-deposition area, forming an enrichment zone [2].

### 3.4. Speciation Characteristics of PTEs in Farmland Soils of Exposed Black Shale Areas

To investigate contamination characteristics of farmlands in exposed black shale areas, systematic sampling was conducted on low-lying paddy soils adjacent to black shale profiles. During 2019–2020, 11 rice–soil paired samples (rice grains and paddy soils) and 8 additional samples were collected in two phases (spatial distribution in Supplementary Material Figure S7). Samples CSD005, CSD006, and CSD015 were collected along tributaries near Huibu Town, whereas CSD008 and CSD012 originated from soils with black shale parent materials containing argillaceous shale and minor carbonate interlayers, exhibiting contamination profiles consistent with pure black shale. Farmlands were ranked by proximity to black shale exposures: GFSDT20-12 ≈ GFSDT20-13 ≈ GFSDT20-15 < GFSDT20-09 ≈ GFSDT20-10 ≈ GFSDT11 < GFSDT20-14 ≈ GFSDT20-08. Among these, GFSDT20-13 and GFSDT20-15 were collected from farmlands <100 m downstream of profiles CS007 (stone coal mining site) and CS003; GFSDT20-11 and GFSDT20-12 were located near road embankments with no adjacent black shale exposures, with GFSDT20-12 situated close to a highway and mountainous terrain.

**Figure 6:**
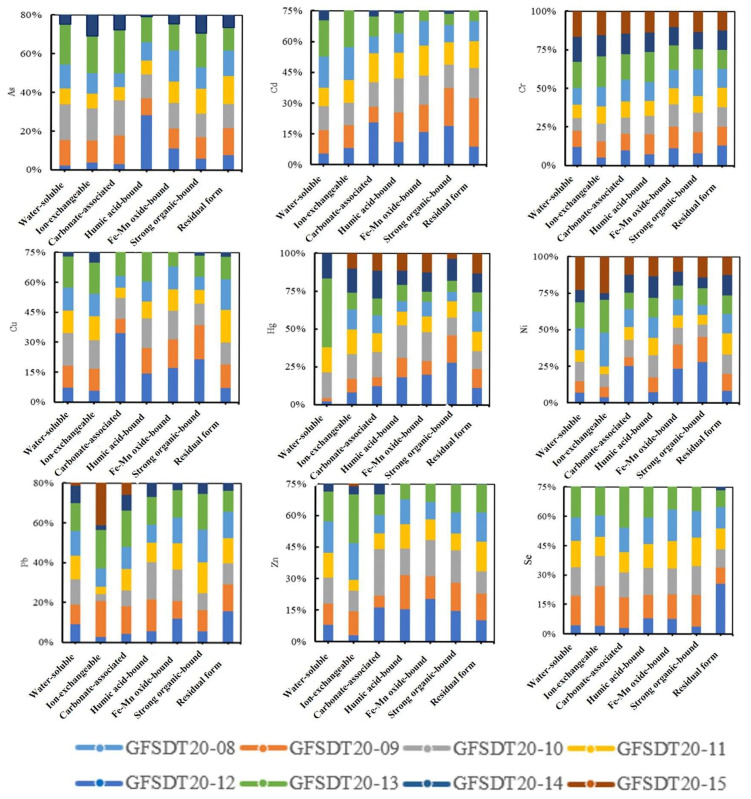
Variations in relative fractions of PTEs and Se in paddy soils from the exposed black shale area of Changshan.

A speciation analysis of Cd, Cr, As, Pb, Hg, Ni, Cu, Zn, and Se was performed on eight 2020 rice samples using the seven-step sequential extraction method (Figure 6). Across all rice soils, the exchangeable fraction constituted the largest proportion of cadmium (27.3–44.2%). Upon removal of this fraction, distinct groupings emerged based on residual properties: Group I (GFSDT20-12/13/15): High bioavailability, characterized by dominant water-soluble Cd. Group II (GFSDT20-09/10/11): Moderate bioavailability, primarily featuring carbonate-bound, humic acid-bound, and Fe-Mn oxide-bound Cd. Group III (GFSDT20-08/14): Stable Cd (predominantly a residual fraction), dominated by strongly organic-bound and residual forms. (Detailed descriptions in Supplementary Material Text S10).

The study demonstrates that Cd speciation strongly correlates with the distance from black shale exposures: increasing migration distance (Group I → II → III) reduces labile fractions; with increasing transport distance, the mobility of Cd progressively decreases, corresponding to a transition from water-soluble forms toward residual fractions. Therefore, Groups I, II, and III can be explicitly classified as proximal, transitional, and distal zones, respectively (see Supplementary Material Text S10 for detailed descriptions).

As shown in Figure 6, based on the classification of farmland proximity to exposed black shale areas, the speciation of other PTEs also undergoes transformation during migration. Detailed multi-element speciation analyses (Cr, As, Pb, Hg, Ni, Cu, Zn, and Se) are provided in Supplementary Material Text S10. Notably, these speciation transformations directly affect heavy metal uptake by rice: ion-exchangeable and water-soluble fractions (dominant in proximal zones) are readily absorbed by rice roots, while stable fractions (e.g., residual and crystalline Fe-oxide-bound forms in distal zones) show a weaker bioavailability, consistent with the lower heavy metal concentrations in rice grains from distal areas

In summary, the strongly acidic, PTE-enriched solutions generated by the weathering of the widely exposed black shale series in western Zhejiang migrate and disperse via hydrological pathways. These processes not only alter the physicochemical properties of watershed soils (including farmlands) and disrupt soil composition, but they also introduce labile PTEs (water-soluble, ion-exchangeable, humic acid-bound, and strongly organic-bound fractions), resulting in geogenic high-background PTE accumulation [2]. Such labile fractions in proximal and transitional zone soils correspond to elevated Cd and As contents in rice (1.2–2.8 mg/kg for Cd, exceeding the national standard (National Health Commission of the PRC/National Food Safety Standard - Maximum Levels of Contaminants in Foods/GB 2762-2022/2022), whereas the higher proportion of carbonate-bound fractions in distal farmlands (surpassing ion-exchangeable fractions) aligns with the lower rice heavy metal levels observed there. Soils in western Zhejiang’s black shale areas exhibit high proportions of labile PTE fractions and a pronounced soil acidification trend. Notably, in farmlands farther from black shale exposures, carbonate-bound fractions even surpass ion-exchangeable fractions in relative abundance. Previous studies report that under acidic conditions, carbonate-bound PTEs can be re-released, posing the risk of secondary contamination [30]. This re-release potential further implies that transitional zone soils, despite lower current rice accumulation, may pose long-term risks to rice safety under acidification (e.g., due to excessive nitrogen fertilizer use).

## 4. Conclusions

This study, employing geochemical analysis and multivariate statistical methods, revealed the migration mechanisms of PTEs in the soils of western Zhejiang’s black shale area. Results show a significant enrichment of Cd and As (3.3× and 2.57× Zhejiang’s background values, respectively), driven primarily by sulfide oxidation and mineral alteration from strongly acidic drainage (pH = 3.9) during black shale weathering [31]. PTEs predominantly existed as active species (e.g., ion-exchangeable and humic acid-bound fractions) during migration, forming a gradient of “highly active near-source accumulation to stable distal transformation [32].” Compared with similar studies, our findings align with the link between black shale weathering and PTE enrichment observed in Yangtze River Delta regions but highlight stronger acidification in western Zhejiang (pH = 3.9) that enhances Cd mobility—with active fractions accounting for over 60% of the total Cd near sources, a higher proportion than reported elsewhere. This regional specificity underscores the role of local geochemical conditions in shaping migration patterns.

Practically, these results support targeted pollution control: neutralizing acidic drainage in near-source zones to reduce active Cd/As release, avoiding soil acidification in transitional areas to prevent secondary pollution, and promoting low-accumulation crops in agricultural regions [31]. Limitations include restricted sampling to Zhuji and Changshan, the potential underestimation of transiently bound fractions, and the lack of climate factor integration, suggesting future work should expand sampling, combine mineralogical analysis, and test tailored remediation strategies. Overall, this study clarifies the unique geochemical behavior of high-background PTEs in black shale regions, providing a theoretical basis for source–pathway, sink-based pollution control to safeguard agricultural safety and ecosystem health [2].

## Figures and Tables

**Table 1:** PTE concentrations in soil from Zhejiang black shale areas (*n* = 5172).

Element	Minimum	Maximum	Mean	SD	Zhejiang Background Value
As (mg kg^−1^)	1.16	688.00	24.74	31.28	6.93
Cd (mg kg^−1^)	0.02	86.90	0.84	2.30	0.25
Cr (mg kg^−1^)	1.08	1380.92	78.20	34.00	79.43
Cu (mg kg^−1^)	5.25	735.00	39.25	29.41	49.68
Hg (mg kg^−1^)	0.01	9.96	0.14	0.19	0.8
Ni (mg kg^−1^)	1.28	364.00	37.60	23.23	31.27
Pb (mg kg^−1^)	11.25	10,273	45.77	170.96	58.06
Zn (mg kg^−1^)	27.70	8089.00	129.01	192.42	124.86

## Data Availability

All available data can be obtained in the Supplementary Materials.

## Supplementary Materials

The following supporting information can be downloaded at: https://www.mdpi.com/article/10.3390/toxics13080679/s1, Text S1: Research Background; Text S2: Regional Geological Overview. Text S3: Black Rock Series Soil Profile Descriptions and Stratification. Text S4: Soil and Crop Sampling, Pretreatment, and Physicochemical Analysis. Text S5: Technical Standards and Specifications. Text S6: Mineral Component Migration and Weathering Characteristics of Weathering Profiles in Typical Black Shale Terranes. Text S7: Explanation of Water–Rock Interactions and Three Element Types (Leached, Illuvial, and Residual) in Western Zhejiang Black Shales. Text S8: Migration States of Heavy Metals in Leached (A, B) Layers and Moderately Weathered (C) Layers of Black Shale Weathering Profiles in Western Zhejiang. Text S9: Geological Background of Cd Enrichment Zones in Quaternary Agricultural Soils Around the Black Shale Terranes in the Changshanjiang River Basin. Text S10 Classification of Type I, II, and III Samples Based on Cd Bioavailability Characteristics and Element Speciation Analysis. Figure S1: Schematic Map of Black Shale Series Distribution in Western Zhejiang. Figure S2: Rock-Weathering Profile Sample Collection. Figure S3: Sampling Location Distribution Map of Black Shale Weathering Profiles in Changshan. Figure S4: Structure of the CS003 Black Shale Weathering Profile. Figure S5: Surface Water Sampling Locations in the Changshan Black Shale Area. Figure S6: Flowchart of Water–Rock Interactions in Black Shales. Figure S7: Distribution Map of Paddy Soil Sampling Locations in the Changshan Black Shale Area. Table S1: Profile Sampling Information. Table S2: Water Sample Information in Changshan Black Shale Area. Table S3: Analytical Methods for Irrigation Water in Black Shale Areas. Table S4: Pearson Correlation Analysis of Heavy Metals in Surface Soils of Zhejiang Black Shale Areas. Table S5-1 Major Element Composition of Black Shale Weathering Profiles in Western Zhejiang (%). Table S5-2: Trace Element Content in Black Shale Weathering Profiles (mg kg-1). Table S6: Trace Element Content Variation Rates (%) at the Rock–Soil Interface of Layer C in Weathering Profiles of Western Zhejiang Black Shales. Table S7: Relative Content (%) of Seven-Step Speciation of Cr, Cd, and As in Soil Layers of CS010 Black Shale Profile. Table S8: Acid Radical Ions and Heavy Metal Content Characteristics of Surface Water in Changshan Black Shale Area. [11,12,13,16,24,25,27,33,34,35,36,37,38,39,40,41,42,43]
