# Supplementary material for: Potentially Toxic Element Migration Characteristics and Bioavailability in Soils of the Black Shale Region, Western Zhejiang Province, China
Source: Toxics. 2025 Aug 14;13(8):679. doi: 10.3390/toxics13080679 (PMC12390234; doi:10.3390/toxics13080679)
Supplement: Supplementary file 1 [file toxics-13-00679-s001.zip › toxics-3764714-supplementary.pdf]

Potentially Toxic Element Migration Characteristics and bioavailability in Soils of the Black Shale Region, Western Zhejiang Province, China—Supplementary Material

Content：Text S1-10

Table S1-7

Figure S1-12

Text S1 Research Background

Zhejiang is a pioneering province in China for conducting 1:250,000 multi-purpose regional geochemical surveys, 1:50,000 soil geochemical surveys, and early-stage heavy metal pollution remediation trials in agricultural lands. From 2002 to 2005, the 1:250,000 Agricultural Geological Environment Survey of Zhejiang Province was conducted, establishing the baseline conditions of agro-geological environments in major agricultural zones. Between 2007 and 2014, 1:50,000 agro-geological surveys were conducted in 15 counties (e.g., Yongkang City) and 14 townships, focusing on geological settings, soil geochemistry, shallow groundwater, agricultural product safety, and specialty crops. Soil geochemical surveys sampled 1–4 sites per km², analyzing eight heavy metals, available nutrients (N, P, K, Fe, Mn, Cu, Zn, Mo, and B), organic matter, Se, and F, enabling agro-geological assessments and zoning.

From 2015 to 2019, the Geochemical Monitoring of Cultivated Land Quality project deployed 2,000 monitoring sites to track dynamic changes in heavy metal pollution, soil acidification, and micronutrient balances. The Zhejiang Land Quality Geological Survey Action Plan (2016–2020) completed 1:50,000 geochemical surveys for 30 million mu (2 million hectares) of farmland across 85 counties, establishing quality archives for 10 million mu (666,667 hectares) of permanent prime farmland, with high-precision data on soil heavy metal variations.

From 2017 to 2019, the former Ministry of Environmental Protection and four

other ministries jointly conducted the National Detailed Survey of Soil Pollution, targeting three priority categories in Zhejiang Province: exceedance zones, key pollution source impact areas, and severely contaminated regions of soil. This survey collected 26,730 surface soil samples, 89 deep soil samples, and 4,099 agricultural product samples. Analytical data included 10 mandatory parameters for surface soils (Cd, Hg, As, Pb, Cr, Cu, Ni, Zn, benzo[a]pyrene, and pH) and five heavy metals (Cd, Hg, As, Pb, and Cr) in matched agricultural products. The results were used to assess soil pollution status and preliminarily classify Zhejiang's soil environmental quality categories.

Additionally, since 2012, the Zhejiang Provincial Department of Agriculture has implemented soil heavy metal pollution surveys and prevention projects in agricultural zones, generating extensive datasets and foundational insights for this study.

Text S2 Regional Geological Overview

The study area (Figure S1) encompasses the western Zhejiang region where black shales are predominantly distributed in Changshan, Kaihua, Jiangshan, Jiande, and Zhuji in northwestern Zhejiang. These shales formed during the Cambrian period under the uplift of the Yangtze Platform, comprising a suite of carbonaceous siliceous argillaceous rocks (containing stone coal) or carbonaceous silicate rock formations (Wang 2022). During the Early Cambrian period, global sea-level rise extensively inundated the carbonate platform of the Dengying Stage. Subsequent large-scale transgressions established a shelf environment dominated by fine-grained siliceous deposits. The rapid transgression preserved pre-existing topography, with the resulting drowned platform controlling subsequent sedimentation. The black shale series are deposited unconformably or conformably over Dengying Stage dolomites or coeval siliceous rocks, and the black shale areas are commonly associated with stone coal and Cu-Pb-Zn polymetallic deposits (Mao et al. 2004). Zhejiang Province, situated on the eastern margin of the Eurasian continent, constitutes a critical component of the Circum-Pacific magmatic belt. Its complete stratigraphic succession spans from the Proterozoic to Quaternary, featuring complex geological structures that straddle two

major tectonic units: the Yangtze Paraplatform and the South China Fold System, demarcated by the Jiangshan-Shaoxing Deep Fault. The crustal evolution involved three major tectonic developmental stages (geosyncline, platform, and continental marginal active belt) and six tectonic cycles (Shenggong, Jinning, Caledonian, Indosinian, Yanshanian, and Himalayan) (Mao et al. 2004, Mao et al. 2005). The stratigraphy of Zhejiang exhibits distinct regional characteristics: northwest Zhejiang belongs to the Jiangnan Stratigraphic Zone, while southeast Zhejiang falls under the South China Stratigraphic Zone, separated by the Jiangshan-Shaoxing Fault. In northwest Zhejiang, Proterozoic to Cenozoic strata are well-developed, with a basement composed of the Neoproterozoic Shuangxiwu and Heshangzhen Groups, overlain by thick Sinian-Cenozoic sedimentary cover. Southeast Zhejiang's metamorphic basement consists of the Paleoproterozoic Badu Group and Mesoproterozoic Chencai and Longquan Groups (Lijun 2012). The Badu Group occurs as the fault block uplifts in the Longquan–Qingyuan, Suichang Datuo, and Songyang Gaoting–Yuyan areas, primarily comprising amphibolite facies regional metamorphic rocks such as plagioclase amphibolite, granulite, and biotite schist. Cretaceous–Cenozoic strata in Zhejiang show no significant regional differentiation: Cretaceous deposits are mainly continental clastic-volcanic sediments in fault basins, while Cenozoic deposits predominantly comprise Quaternary alluvial, diluvial, and coastal loose sediments.

Magmatic activity in the study area is frequent and dominated by Yanshanian intrusions, with minor Shennongian, Jinningian, Indosinian, and Himalayan phases. Intrusions primarily occur as stocks and dikes, dominated by acidic/intermediate-acidic rocks (granite, alkaline granite, and monzogranite) (Wang et al. 2007).

Bedrock fundamentally controls the soil elemental composition. Soil geochemical characteristics strongly correlate with parent rock geochemistry (Wang et al. 2007). Major soil-forming rocks in Zhejiang exhibit distinct elemental differences between mountainous/hilly and plain regions. Multi-target geochemical survey data reveal the following: MgO, CaO, Na2O, N, P, Cl, Mn, Cu, B, Ba, Li, Sn, Sr, Cr, Hg, Ni, F, and available B/Cu/Mn/Zn/Fe are enriched in plains; Mo, Se, Br, I, Sb, U, As, and available

Mo are higher in mountainous areas. MgO, CaO, Na2O, and Cl enrich marine/fluvial parent materials, while Mo, Se, Br, I, Sb, U, and As concentrate in Paleozoic sedimentary/volcanic/granitic rocks. N, P, and B variations stem from agricultural fertilization and organic matter content, which positively correlates with these elements. Cr, Hg, Ni, and F enrichment in plains may result from surface processes and anthropogenic pollution (Wang 2022).

The study area displays diverse geomorphological and bioclimatic conditions, coupled with long-term agricultural development, creating distinct soil zonation. Zhejiang soils can be categorized into four types based on geomorphology: coastal tidal flats, alluvial plains, valley basins, and hilly/mountainous regions (Lijun 2012). Western Zhejiang hilly soils are dominated by red earth, yellow earth, and paddy soils, with local calcareous soils.

Text S3 Black Rock Series Soil Profile Descriptions and Stratification

CS003: Located at Caili Village, Changshan County, this Hetang Formation black shale weathering profile collected eight samples (two soils, six rocks, including two weathered gravels). Situated on a hilltop within a folded anticline, the weathering intensity increases from core to limbs. The core contains stone coal, transitioning to yellowish rocks with increasing soil transformation upward. Thin weathering layers underlie vegetation-covered upper slopes with large trees.

CS009: At Shibali Village, Changshan County, this Hetang Formation black shale profile collected nine samples (five soils, four rocks). Thick yellow-brown weathering layers overlie moderately weathered yellowish shale, darkening downward with less weathering at depth. Upper slopes support dense vegetation.

CS010: Xinan Village, Changshan County, Hetang Formation black shale profile with 12 samples (four soils, eight rocks). Thin yellow-brown topsoil overlies semi-weathered shale and fresh bedrock (black shale with stone coal). Sparse vegetation and underdeveloped root systems characterize upper slopes.

Profile Stratification (A–E layers, Figure S4):

A (Topsoil): Plant-rooted, homogeneous soil with small gravel, high clay/secondary minerals.

B (Strongly weathered): Porous, loose texture with abundant free oxygen/water

enabling intense oxidation/hydrolysis. Larger gravel than A.

C (Moderately weathered): Compact structure transitioning to the rock–soil interface. Primary bedding disappears due to oxidation/hydrolysis. Contains mixed soil–rock fragments with gray-yellow coloration from differential weathering.

D (Weakly weathered): Slightly oxidized bedrock with gray-black/gray-blue color, retaining original structure/bedding.

E (Bedrock/stone coal): Massive/schistose, dense rocks with pungent odor, staining hands.

Text S4 Soil and Crop Sampling, Pretreatment, and Physicochemical Analysis

Soil Sampling Method: All sampling points were located using GPS. Soil samples were collected at a depth of 0–20 cm. At the sampling site, soil samples were initially cleaned of debris, placed into cloth bags, air-dried, and then further cleaned of plant roots, insects, stones, and other impurities before being crushed with a rubber hammer and sent to the laboratory for analysis. Rice grains were threshed, air-dried, and subsampled using the quartering method. After laboratory processing into rice samples, they were subjected to analysis.

Sample Pretreatment: Following the relevant standards and literature methods (Kalnicky & Singhvi 2001, Shukui & Yongmei 2015), soil samples were dried under constant temperature conditions (<60°C) for chemical analysis. Plant samples were washed, chopped using a specialized shredder, and further pulverized with a contamination-free grinder to 20–40 mesh (0.84–0.42 mm), sieved, dried, and digested for analysis.

pH: Measured using an electrode: 10.00 g of the original sample was weighed into a 50 mL beaker, mixed with 25 mL of CO_2-free distilled water, stirred, and then measured.

Cd, Cu, Ni, and Sc: Analyzed by inductively coupled plasma mass spectrometry (ICP-MS) (Thermo X Series II, Thermo Electron Corporation). A 0.1000–0.2500 g sample was digested with hydrofluoric acid, nitric acid, and perchloric acid on a 150°C hotplate for over 8 hours in a sealed container. After cooling, the container was opened,

and the hotplate temperature was raised to 180°C to evaporate residual perchloric acid. The residue was dissolved in nitric acid, transferred to a polyethylene tube, and diluted to 25 mL. After shaking and clarification, a portion of the supernatant was diluted with (3+97) nitric acid to achieve a total dilution factor of 1000. Analysis was performed using Rh and Ir as dual internal standards on the ICP-MS.

Pb, Zn: Determined by X-ray fluorescence spectroscopy (XRF) (ZSX100e, Rigaku Corporation, Japan). Samples were dried in a 105°C oven, cooled, and approximately 4 g of the sample was pressed into a plastic ring under 10 MPa pressure to form a pellet (outer diameter: 40 mm; inner diameter: 32 mm). Automatic sample introduction was used with empirical coefficient and scattered radiation internal standard methods to correct for absorption enhancement effects between elements. Multi-element determination was performed using the XRF spectrometer.

As: Analyzed by hydride generation atomic fluorescence spectrometry (HG-AFS). A 0.2500 g sample was weighed into a 25 mL test tube, digested with (1+1) aqua regia in a 100°C water bath for 1 hour, diluted to volume, and shaken. An aliquot was taken, reduced with thiourea-ascorbic acid, and analyzed using potassium borohydride as the reductant via HG-AFS.

Hg: Determined by cold vapor atomic fluorescence spectrometry (CV-AFS). A 0.5000 g sample was digested with (1+1) aqua regia in a 100°C water bath for 1 hour. After cooling, potassium permanganate solution was added and allowed to stand for 30 minutes, followed by oxalic acid solution. The solution was diluted to volume and shaken. Using a mercury high-intensity hollow cathode lamp and stannous chloride as the reductant, Hg^{2+} was reduced to Hg vapor, which was carried by argon into a preheated 200°C quartz atomizer (XGY-1011A) for CV-AFS measurement.

Organic Matter: Analyzed by high-frequency heating infrared absorption. A 2–10 mg sample was placed in a small quartz cup, treated with dilute hydrochloric acid to remove inorganic carbon, dried, and then analyzed using high-frequency heating infrared absorption. National primary soil standard materials were analyzed under

identical conditions to establish calibration curves for calculating organic carbon content. Quality control was ensured through duplicate subsample testing and comparison with certified reference materials, with differences between measured and standard values required to be less than 5%. The precision (RSD%) of reference materials ranged from 1.7% to 9.95%.

Text S5 Technical Standards and Specifications

Based on the primary scope of this study, the relevant monitoring and survey protocols, analytical testing methods, and evaluation criteria primarily encompass the following aspects:

1. Technical Requirements for Geochemical Monitoring of Land Quality (DD 2014-10);
2. Technical Rules for Monitoring of Environmental Quality of Farmland Soil (NY/T 395-2012);
3. Technical Specifications Requirements for Monitoring of Surface Water and Waste Water (HJ/T 91-2002);
4. Specification of Land Quality Geochemical Assessment (DZ/T 0295-2016);
5. Determination of 12 Metal Elements in Soil and Sediment by Aqua Regia Extraction Inductively Coupled Plasma Mass Spectrometry (HJ 803-2016);
6. Sequential Extraction Procedure of Speciation of 13 Trace Elements in Soil and Sediment (GB/T 25282-2010);
7. National Food Safety Standard—Determination of Multi-elements in Foods (GB 5009.268-2016);
8. Determination of 65 Elements in Water by Inductively Coupled Plasma-Mass Spectrometry (HJ 700-2014);
9. Analytical Methods and Technical Requirements for Eco-geochemical Evaluation Samples (DD 2005-03);
10. Risk Control Standard for Soil Contamination of Agricultural Land (Trial) (GB 15618—2018);
11. Standards for Irrigation Water Quality (GB 5084-2005);

12. National Food Safety Standard—Maximum Levels of Contaminants in Foods (GB 2762-2017).

Text S6 Mineral Component Migration and Weathering Characteristics of Weathering Profiles in Typical Black Shale Terranes

Black shales are primarily composed of quartz, clay minerals, potassium feldspar, sulfide minerals, organic matter, and minor carbonate minerals, all of which (except quartz) are prone to decomposition under supergene conditions. As shown in Table S4, the weathering process of black shales exhibits four distinct stages:

1. From the bedrock layer (E) to the weakly weathered layer (D), SiO_2 content increases significantly, while alkaline components such as CaO and MgO are rapidly leached due to carbonate mineral weathering, consistent with the preferential dissolution model of carbonates proposed by Arthur & Susan (1995).

2. During the transition from layer D to the moderately weathered layer (C), alkaline components further decrease, and Al_2O_3 gradually becomes enriched. This aligns with the de-alkalization process described by Nesbitt & Young (1984), where Al_2O_3 enrichment is attributed to the formation of clay minerals (e.g., kaolinite).

3. In the transition from the moderately weathered layer (C) to the strongly weathered soil layer (B), abrupt changes in major element concentrations occur (e.g., CaO in profile CS003 drops from 8.25% to 0.05%). This indicates intense acidification and hydrolysis in the leaching layer, leading to the extensive decomposition of carbonate minerals and significant loss of cations (Ca, Mg, Na, and K), replaced by Al, Fe, and Mn. The relative concentrations of major elements stabilize here, a phenomenon matching the "weathering front" features observed by Brantley & Lebedeva (2011) in black shale regions. Their model confirms that H^+ diffusion-controlled carbonate dissolution drives Ca^{2+} and Mg^{2+} migration (flux up to 10^{-10} mol/m²/s). The substitution by Al^{3+} and Fe^{3+} stabilizes elemental composition, resembling the Al-Fe oxide-dominated phase in granite weathering profiles reported by Brantley et al. (2007).

4. From the strongly weathered layer (B) to the topsoil (A), elements such as K, Na,

Mg, and Ca show slight increases due to plant uptake and biogeochemical cycling. This may relate to nutrient transport from deeper layers to the surface via root-mediated cation exchange, causing re-enrichment of alkali and alkaline earth metals in topsoil (Jobbágy & Jackson 2004).

The degree of profile weathering is influenced by multiple factors, including topography, groundwater table, pH, and oxygen content of groundwater, rock permeability, mineral composition, rock structure, fracture development, vegetation roots, microbes, and climatic conditions (Dixon & von Blanckenburg 2012). The Chemical Index of Alteration (CIA) and concentrations of metal oxides (e.g., CaO, MgO, and Na_2O) are commonly used to investigate the weathering intensity and elemental migration mechanisms in black shale systems (Nesbitt & Young 1984). Key weathering characteristics of the profiles are as follows:

- **CS003 Profile** (CIA: 65.27–83.44): High CIA in bedrock suggests prolonged supergene exposure (Nesbitt & Young 1984). CIA drops sharply in the weakly weathered layer due to high CaO (carbonate enrichment), while moderate Al_2O_3, MgO, CaO, and Na_2O levels indicate relatively low weathering intensity (Arthur & Susan 1995).
- **CS009 Profile** (CIA: 59.58–83.18): Low Al_2O_3 and high MgO/Na_2O in topsoil and strongly weathered layers suggest weaker weathering compared to CS010.
- **CS010 Profile** (CIA: 56.21–87.70): Highest Al_2O_3 and lowest mobile oxides (MgO, CaO, and Na_2O) reflect the most advanced leaching and weathering (Brantley & Lebedeva 2011).

Comparisons of CIA and Rc (residual coefficient) across profiles reveal that chemical weathering predominantly occurs from the weakly weathered layer (D) to the strongly weathered layer (B). A notable elemental abrupt transition occurs at the rock–soil interface in the moderately weathered layer (C), characterized by rapid CaO depletion and Na enrichment, linked to the kinetic preferential dissolution of carbonates, Bufe et al. (2021). In CS003, intense acidification and hydrolysis in the moderately to

strongly weathered layers lead to massive MgO, CaO, Na_2O, and K_2O loss, driving CIA and Rc to peak values (Bufe et al. 2021). Changes in major elements from strongly weathered layers to topsoil are minimal. Notably, gravels sieved from CS003 and CS010 topsoil exhibit high MgO/CaO, low Na_2O/K_2O, and low SiO_2/Al_2O_3, indicating low CIA values compared to the parent rock but higher than those in the moderately weathered layer (C), reflecting residual weathering products undergoing further alteration in the soil (Li et al. 2024). Overall, deepening weathering correlates with rapid CaO/MgO depletion and progressive Al/Fe oxide enrichment, highlighting de-alkalization, ferro-aluminization, and intensified acidification.

Text S7 Explanation of Water–Rock Interactions and Three Element Types (Leached, Illuvial, and Residual) in Western Zhejiang Black Shales

Water–rock interactions: As illustrated in Figure S6, when sulfide-rich black shales are exposed to surface air, oxidative hydrolysis generates acidic water enriched with Fe and S. This acidic water reacts with feldspar and clay minerals in the parent rock, causing clay mineral expansion. This process increases rock permeability and alters internal cementation, modifying the shale's physical properties (Lin et al. 2017). Concurrently, organic matter in black shales oxidizes through self-decomposition or interactions with sulfide-derived acidic water, releasing CO_2 and increasing H^+ concentration, thereby accelerating silicate mineral dissolution (Wu et al. 2020). At the rock–soil interface, the rapid depletion of CaO and MgO minerals occurs alongside significant shifts in trace element concentrations. Overall, the chemical weathering of black shales exhibits cyclic progression, characterized by the leaching of elements like Ni, Cu, Zn, Cd, and Se, and enrichment of TC, Cr, and Hg (Wei et al. 2025).

The explanation of leached, illuvial, and residual elements:

1. Leached elements are those permanently removed from the weathering profile without re-enrichment.
2. Illuvial elements migrate from the upper topsoil and strongly weathered layers (A/B) to accumulate in the moderately weathered layer (C).
3. Residual elements show negligible mobility during weathering.

Compared to bedrock, illuvial elements exhibit enrichment in layer C but depletion in layers D, A, and B. Leached elements remain in a net depletion state, with pronounced losses in layer C due to dilution effects from illuvial element accumulation. Residual elements are enriched in layers A, B, and D. In layer C, their enrichment is diminished or even reversed by combined effects of concentration (from leaching) and dilution (from illuvial and leached elements).

Text S8 Migration States of Heavy Metals in Leached (A, B) Layers and Moderately Weathered (C) Layers of Black Shale Weathering Profiles in Western Zhejiang

1. **Cr**: Chromium primarily exists in the residual fraction, accounting for an average of 90.68% of total Cr content in the soil. The water-soluble and ion-exchangeable fractions are low, with contents in all layers below 0.5%. The carbonate-bound, humic acid-bound, and iron-manganese oxide-bound fractions are highest in layer B (strongly weathered leached zone), and their contents in layer A (topsoil leached zone) are higher than in layer C (moderately weathered layer). The strong organic-bound fraction shows a cumulative enrichment trend with increasing weathering intensity.
2. **Cd**: Cadmium primarily exists in ion-exchangeable, humic acid-bound, and residual fractions. The water-soluble, carbonate-bound, iron-manganese oxide-bound, and strong organic-bound fractions are significantly depleted. As weathering intensifies (from layer C to A), the proportion of the water-soluble fraction first increases and then decreases, the ion-exchangeable fraction decreases and then rises, the carbonate-bound fraction shows cumulative enrichment, the humic acid-bound fraction gradually depletes, the iron-manganese oxide-bound fraction accumulates, the proportion of the strong organic-bound fraction slightly increases, and the residual fraction rises rapidly before slightly declining.
3. **As**: Arsenic exhibits a distribution pattern similar to Cr, with the residual fraction dominating (averaging 88.21% of total As content in the soil). The water-soluble, ion-exchangeable, carbonate-bound, and strong organic-bound fractions are all below 0.5%. As weathering intensifies (from layer C to A), the proportion of the

humic acid-bound fraction slightly decreases before rising, forming enrichment in the topsoil, while the iron-manganese oxide-bound fraction declines with increasing weathering intensity.

Text S9 Geological Background of Cd Enrichment Zones in Quaternary Agricultural Soils Around the Black Shale Terranes in the Changshanjiang River Basin

Areas a and b are artificially excavated sites. Area a is a local coal mining site in Changshan, where the collected strata belong to the Lower Cambrian Hetang Formation. Area b is a construction excavation site. The exposed surface strata in area b are the Xihu Formation and Zhucangwu Formation, with the underlying strata being the Hetang Formation. The upper part of the Zhucangwu Formation consists of purple sandstone, mudstone, and gray-black sandstone interbedded with mudstone and coal seams, also containing the black shale series. Area c corresponds to the Hejia Village-Changshan County township zone, featuring no large-scale excavation areas overall, with only small local excavations far from water bodies, thus classified as non-excavated areas. Area d is located at the urban fringe with relatively high elevation, no exposed bedrock surfaces, and subsurface strata belonging to the Hetang Formation.

Text S10 Classification of Type I, II, and III Samples Based on Cd Bioavailability Characteristics and Element Speciation Analysis

Type I (GFSDT20-12/13/15; High Bioavailability Characteristics): High ion-exchangeable fraction (55%–62%), high water-soluble fraction (0.71%–1.03%), with lower proportions of carbonate-bound (12%–14%), humic acid-bound (8%), iron-manganese oxide-bound (10%–16%), strongly organic-bound (2%–4%), and residual fractions (3%–6%).

Type II (GFSDT20-09/10/11; Moderate Bioavailability Characteristics): Medium ion-exchangeable fraction (37%–38%), medium water-soluble fraction (0.42%–0.54%), with transitional distributions of carbonate-bound (11%–20%), humic acid-bound (10%–14%), iron-manganese oxide-bound (18%–19%), strongly organic-bound (4%–6%), and residual fractions (8%–14%).

Type III (GFSDT20-08/14; Approaching Cd Stable State): Low ion-exchangeable

fraction (37%–38%), low water-soluble fraction (0.26%–0.36%), high carbonate-bound fraction (28%–30%), and increased proportions of humic acid-bound (9%–13%), iron-manganese oxide-bound (17%–21%), strongly organic-bound (5%–6%), and residual fractions (5%–10%).

The parent materials of Type I, II, and III samples are all derived from the Hetang Formation black shale series, located far from residential and industrial areas, allowing negligible anthropogenic interference. The Cd in farmland soils exhibits similar initial speciation ratios. Therefore, the primary factor driving changes in Cd speciation is the strongly acidic heavy metal leachate from weathering profiles in black shale-exposed areas. Based on the trends of Cd speciation in black shale weathering profiles, the three sample types are analyzed as follows:

Type I: Close to black shale exposures and influenced by strongly acidic heavy metal solutions, the farmland soils exhibit relatively high degradation of carbonate minerals, humic acids, iron-manganese oxides, and organic matter. Heavy metal pollutants primarily exist in water-soluble and ion-exchangeable forms, with low residual fraction proportions. Consequently, water-soluble fractions are saturated (lower than in weathering profile soils), ion-exchangeable fractions are dominant, and other forms are depleted.

Type II: As the acidic solution loses acidity through partial neutralization during migration, its impact on carbonate minerals, humic acids, iron-manganese oxides, and organic matter in farmland soils weakens. However, carbonate mineral content is generally high in Changshan's exposed strata, and iron-manganese oxide content increases. Despite reduced organic matter degradation, humic acid-bound and strongly organic-bound fractions remain depleted. The ion-exchangeable and water-soluble fractions decrease during migration, but the ion-exchangeable fraction still dominates due to its initially large proportion. The total Cd content declines slightly, and the residual fraction shows a relative increase.

Type III: As the acidic heavy metal solution migrates further, its acidity decreases,

further reducing damage to carbonate minerals, humic acids, iron-manganese oxides, and organic matter. The ion-exchangeable and water-soluble fractions drop significantly. Due to the relatively high carbonate mineral content in Changshan's black shale area, the carbonate-bound fraction becomes dominant. Humic acid-bound and iron-manganese oxide-bound fractions rise rapidly, the strongly organic-bound fraction remains depleted, and the residual fraction stabilizes.

Element Speciation Composition Analysis:

Cr: The overall speciation of Cr is similar to that of the weathering profile soil layers, with the residual fraction being the dominant form. As migration distance increases, the relative proportions of the strongly organic-bound, iron-manganese oxide-bound, and humic acid-bound fractions show a slight depletion.

As: The overall speciation of As resembles that of the weathering profile soil layers, also dominated by the residual fraction. With increasing migration distance, the relative proportions of the humic acid-bound and iron-manganese oxide-bound fractions gradually decline. The carbonate-bound fraction is consistently depleted, and its depletion intensifies with greater migration distances.

Pb: Pb primarily exists in the residual fraction, followed by iron-manganese oxide-bound, carbonate-bound, and humic acid-bound fractions, while the strongly organic-bound, water-soluble, and ion-exchangeable fractions are depleted. As migration distance increases, the relative proportions of the residual and iron-manganese oxide-bound fractions accumulate, whereas the carbonate-bound and humic acid-bound fractions show depletion.

Hg: Hg is mainly present in the residual fraction, followed by humic acid-bound and strongly organic-bound fractions. As migration distance increases, the residual fraction gradually depletes, while the humic acid-bound and strongly organic-bound fractions accumulate.

Ni: Ni primarily exists in the residual fraction, followed by iron-manganese oxide-

bound and strongly organic-bound fractions. With increasing migration distance, the residual and iron-manganese oxide-bound fractions accumulate, whereas the strongly organic-bound fraction shows no significant trend, and the ion-exchangeable fraction gradually depletes.

Cu: Cu is predominantly in the residual fraction, followed by humic acid-bound, iron-manganese oxide-bound, strongly organic-bound, and carbonate-bound fractions. As migration distance increases, the residual fraction accumulates, while the humic acid-bound and carbonate-bound fractions deplete. The proportions of iron-manganese oxide-bound and strongly organic-bound fractions remain unchanged.

Zn: Zn mainly exists in the residual fraction, followed by iron-manganese oxide-bound, humic acid-bound, carbonate-bound, strongly organic-bound, and ion-exchangeable fractions. With increasing migration distance, the residual and ion-exchangeable fractions deplete, whereas the carbonate-bound, humic acid-bound, and iron-manganese oxide-bound fractions accumulate. The strongly organic-bound fraction remains stable without noticeable trends.

Se: Se predominantly occurs in the strongly organic-bound fraction, followed by residual and humic acid-bound fractions, with minor enrichment in the carbonate-bound fraction. All other forms of Se are depleted. As migration distance increases, the residual and strongly organic-bound fractions accumulate, while the humic acid-bound and carbonate-bound fractions gradually deplete.

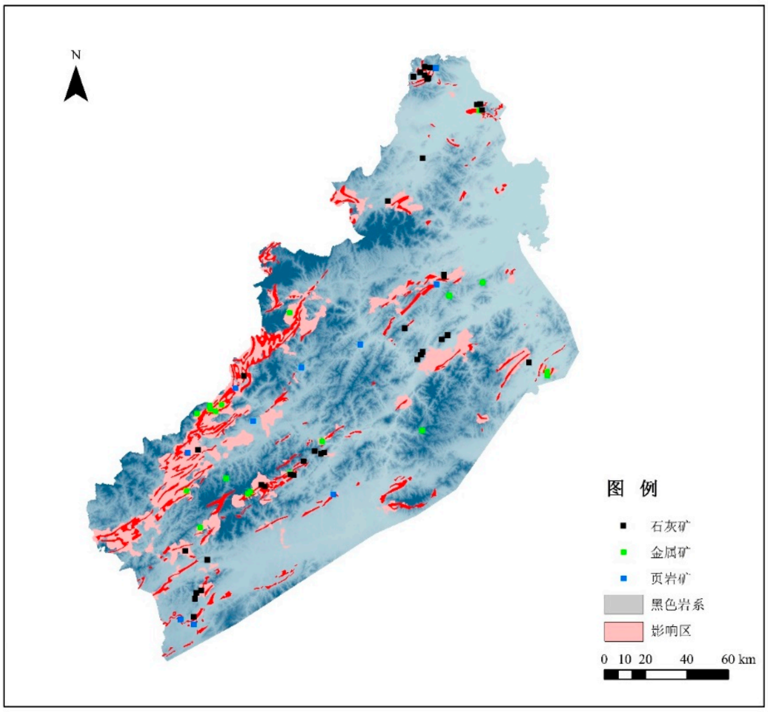

Figure S1 Schematic Map of Black Shale Series Distribution in Western Zhejiang

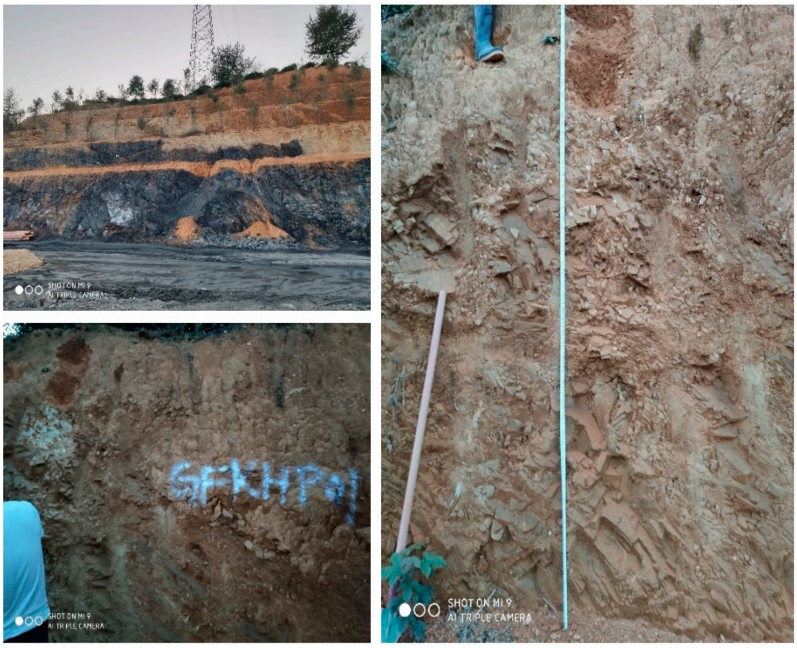

Figure S2 Rock-Weathering Profile Sample Collection

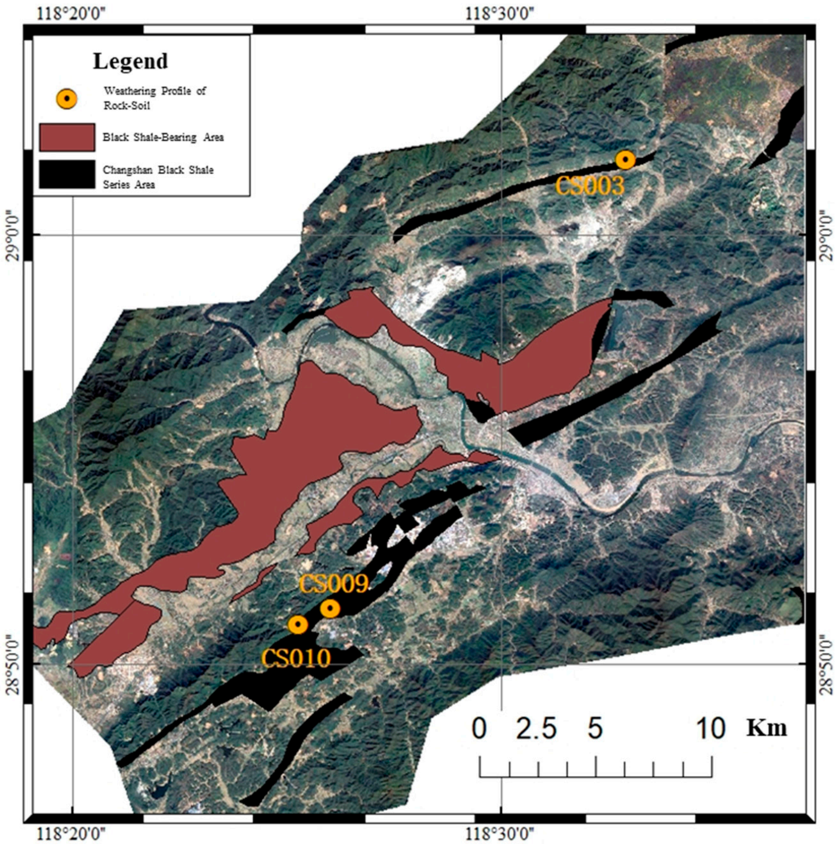

Figure S3 Sampling Location Distribution Map of Black Shale Weathering Profiles in Changshan

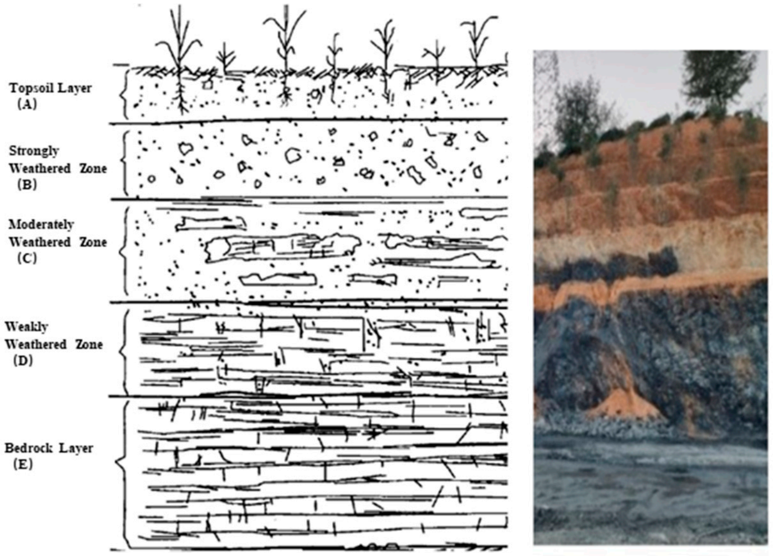

Figure S4 Structure of the CS003 Black Shale Weathering Profile

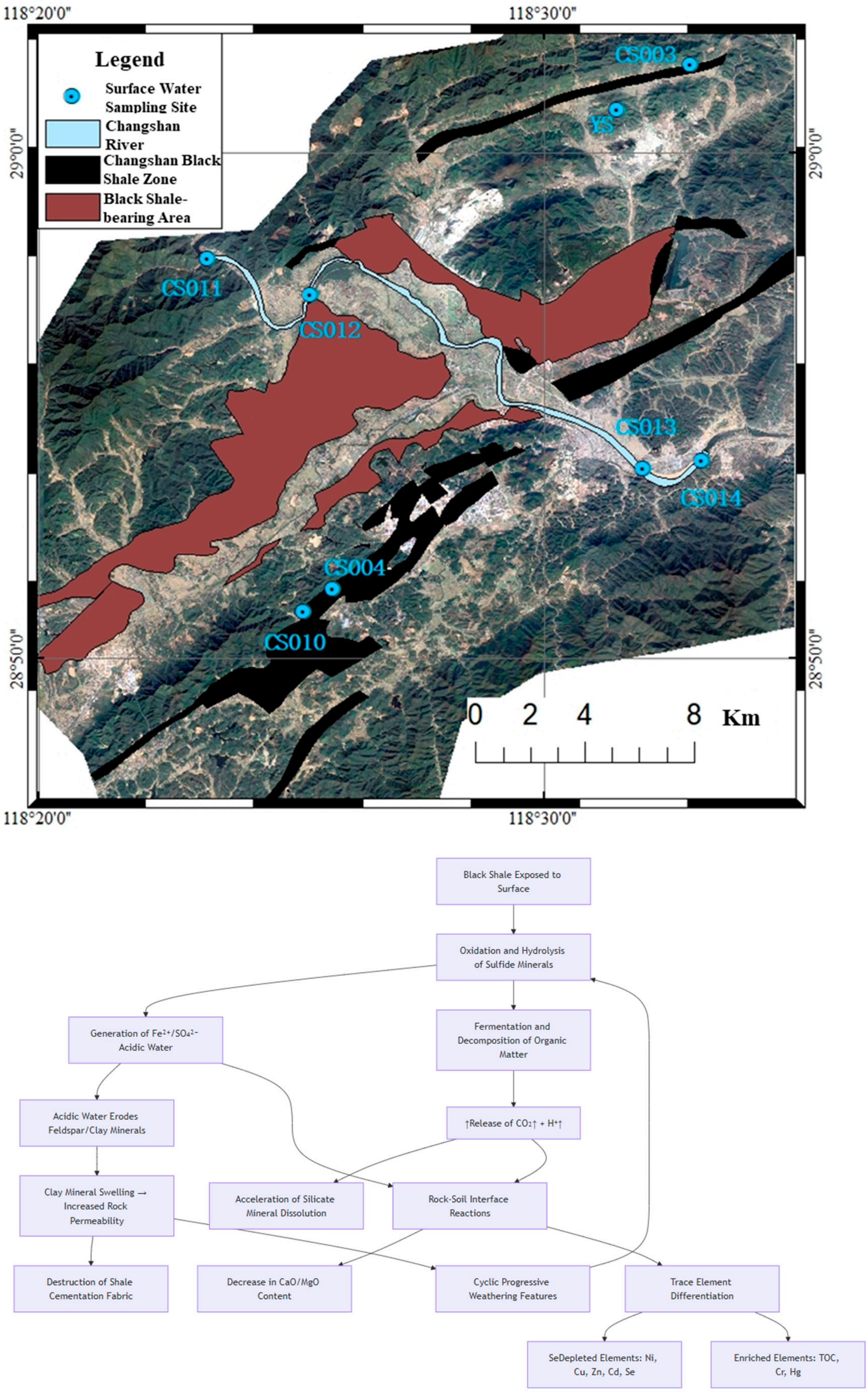

Figure S5 Surface Water Sampling Locations in the Changshan Black Shale Area.

Figure S6 Flowchart of Water–Rock Interactions in Black Shales

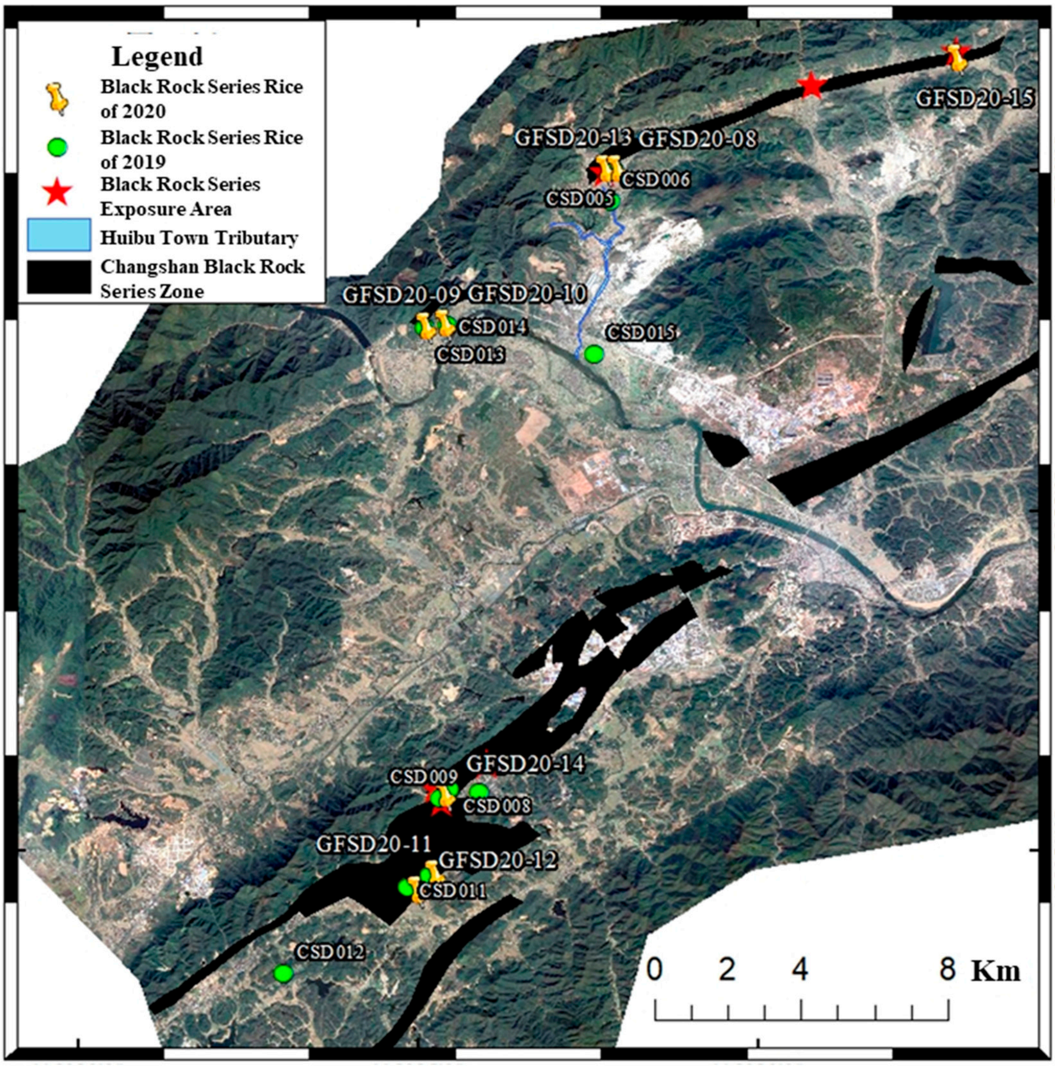

Figure S7 Distribution Map of Paddy Soil Sampling Locations in the Changshan Black Shale Area (*Note: The width of the tributary channel in Huibu Town has been widened for illustrative purposes, indicating relative positions rather than actual dimensions.*)

Table S1 Profile Sampling Information

No.	Point ID	Location	Elevation (m)	Landform	Sample Lithology
1	CS003	Caili Village Coal Mine	201	Mountaintop	Fresh Stone Coal
2	CS003	Caili Village Coal Mine	201	Mountaintop	Surface Stone Coal with Brick-Red Material
3	CS003	Caili Village Coal Mine	201	Mountaintop	Purple-Red Shale (First-Level Weathering Product)
4	CS003	Caili Village Coal Mine	201	Mountaintop	Purple, Red, and Gray-Yellow Shale (Second-Level Weathering Product)
5	CS003	Caili Village Coal Mine	201	Mountaintop	Gray-Yellow Shale (Third-Level Weathering Product)
6	CS003	Caili Village Coal Mine	201	Mountaintop	Gray-White Shale (Fourth-Level Weathering Product)
7	CS003	Caili Village Coal Mine	201	Mountaintop	Gray-Yellow Soil (High Sand-Gravel Content)
8	CS003	Caili Village Coal Mine	201	Mountaintop	Weathered Soil with Gravel
9	CS003	Caili Village Coal Mine	201	Mountaintop	Gray-Yellow Soil (High Sand-Gravel Content)
10	CS003	Caili Village Coal Mine	201	Mountaintop	Weathered Soil with Gravel
11	CS009	Shibali Village Woodland	141	Mountaintop	Fresh Black Shale
12	CS009	Shibali Village Woodland	141	Mountaintop	Black Shale (First-Level Weathering Product)
13	CS009	Shibali Village Woodland	141	Mountaintop	Black Shale (Third-Level Weathering Product)
14	CS009	Shibali Village Woodland	141	Mountaintop	Black Shale (Second-Level Weathering Product)
15	CS009	Shibali Village Woodland	141	Mountaintop	Residual Soil
16	CS009	Shibali Village Woodland	141	Mountaintop	Residual Soil
17	CS009	Shibali Village Woodland	141	Mountaintop	Residual Soil
18	CS009	Shibali Village Woodland	141	Mountaintop	Residual Soil
19	CS009	Shibali Village Woodland	141	Mountaintop	Residual Soil
20	CS010	Xin'an Village Coal Mine	158	Mountaintop	Fresh Black Shale (Stains Hands)
21	CS010	Xin'an Village Coal Mine	158	Mountaintop	Black Shale (First-Level Weathering Product)
22	CS010	Xin'an Village Coal Mine	158	Mountaintop	Black Shale (Second-Level Weathering Product)
23	CS010	Xin'an Village Coal Mine	158	Mountaintop	Black Shale (Third-Level Weathering Product)
24	CS010	Xin'an Village Coal Mine	158	Mountaintop	Residual Soil
25	CS010	Xin'an Village Coal Mine	158	Mountaintop	Residual Soil
26	CS010	Xin'an Village Coal Mine	158	Mountaintop	Weathered Soil with Gravel
27	CS010	Xin'an Village Coal Mine	158	Mountaintop	Residual Soil
28	CS010	Xin'an Village Coal Mine	158	Mountaintop	Weathered Soil with Gravel
29	CS010	Xin'an Village Coal Mine	158	Mountaintop	Residual Soil

Table S2 Water Sample Information in Changshan Black Shale Area

Sample ID	Category	Sample Characteristics
YS	Rainwater	Atmospheric Precipitation
CS003	Mine Wastewater	Wastewater from CS003 Profile
CS004	Mountain Stream	Stream Water from Non-Black Shale Area (Irrigation Water)
CS010	Mine Wastewater	Wastewater from CS010 Profile
CS011	Upstream River	Qiantang River Upstream (Kaihua-Changshan Boundary)
CS012	Upstream River	Qiantang River Upstream
CS013	Downstream River	Qiantang River Downstream
CS014	Downstream River	Qiantang River Downstream

Table S3 Analytical Methods for Irrigation Water in Black Shale Areas

Element/Parameter	Pretreatment	Analytical Method
As, Cd, Cu, Hg, Pb	Acidified Sample	ICP-MS
Zn	Acidified Sample	ICP-OES
Cr(VI)	Alkaline Sample	Colorimetry (COL)
pH	Raw Sample	Glass electrode method
NO_3^-	Raw Sample	COL
NO_2^-	Raw Sample	COL
SO_4^{2-}	Raw Sample	Turbidimetry
Cations (Na^+, Ca^{2+}, K^+, etc.)	Raw Sample	Ion chromatography or atomic Absorption spectrometry
Anions (Cl^-, F^-, SO_4^{2-}, HCO_3^-, etc.)	Raw Sample	Ion chromatography

Table S4 Pearson Correlation Analysis of Heavy Metals in Surface Soils of Zhejiang Black Shale Areas

	As	B	Cd	Co	Cr	Cu	Hg	K_2O	Mn	Mo	N	Ni	P	Pb	Se	V	Zn	SOM
**As**	1																	
**B**	.144**	1																
**Cd**	.188**	0.024	1															
**Co**	.261**	.241**	.127**	1														
**Cr**	.222**	.181**	.088**	.371**	1													
**Cu**	.313**	.137**	.431**	.360**	.356**	1												
**Hg**	.154**	0.004	.049**	-0.012	.075**	.040**	1											
**K_2O**	.093**	0.008	0.011	.224**	-.033*	.128**	-.098**	1										
**Mn**	.284**	-0.026	.482**	.288**	.086**	.230**	.034*	0.001	1									
**Mo**	.270**	.141**	.164**	.136**	.302**	.361**	.089**	.043**	0.02	1								
**N**	-.039**	.161**	.087**	.032*	.044**	.127**	.054**	.101**	-.090**	.033*	1							
**Ni**	.313**	.209**	.430**	.538**	.539**	.562**	.095**	.142**	.207**	.537**	.119**	1						
**P**	.129**	.083**	.118**	.144**	.219**	.315**	.150**	.150**	.087**	.156**	.304**	.281**	1					
**Pb**	.241**	-0.024	.569**	.052**	0.006	.359**	0.025	-0.026	.791**	.031*	-0.015	.126**	.038**	1				
**Se**	.210**	.100**	.301**	0.015	.375**	.332**	.102**	-.066**	.115**	.473**	.205**	.397**	.150**	.182**	1			
**V**	.288**	.239**	.197**	.262**	.509**	.496**	.076**	.103**	.032*	.660**	.058**	.581**	.296**	0.009	.538**	1		
**Zn**	.265**	0.011	.633**	.158**	.379**	.559**	.048**	.042**	.586**	.165**	.036**	.422**	.164**	.726**	.249**	.188**	1	
**SOM**	-.046**	0	.145**	-.093**	-0.011	.068**	.057**	-.034*	-.151**	.056**	.792**	.069**	.265**	.032*	.214**	0.021	.082**	1

*Notes: **p < 0.01; *p < 0.05.*

Table S5-1 Major Element Composition of Black Shale Weathering Profiles in Western Zhejiang (%)

Profile	Layer	Sample Type	SiO_2	Al_2O_3	K_2O	Na_2O	CaO	MgO	CIA	Rc
CS003	A	Soil	82.56	10.94	2.05	0.10	0.05	0.73	83.27	1.05
		Gravel	79.75	7.64	1.83	0.07	1.17	1.49	71.37	1.29
	B	Soil	82.99	10.56	1.95	0.10	0.05	0.68	83.44	1.07
		Gravel	84.20	6.17	1.59	0.05	1.64	1.53	65.27	1.00
	C	Rock	85.78	6.36	1.66	0.07	0.40	0.61	74.81	1.22
	D	Rock	82.98	5.15	1.76	0.11	8.25	0.66	33.92	0.39
	E	Rock	78.29	10.59	2.75	0.34	0.63	2.55	73.98	0.91
CS009	A	Soil	83.74	8.89	1.65	0.11	0.04	0.80	83.18	1.19
	B	Soil	81.26	10.34	1.57	0.11	0.03	0.80	85.69	1.26
	C	Rock-Soil	78.15	12.06	2.56	0.10	0.17	1.23	81.29	1.12
	D	Rock	79.05	9.85	2.97	0.09	1.91	1.37	66.75	0.92
	D	Rock	72.36	12.14	4.42	0.13	3.68	1.74	59.58	0.80
CS010	A	Soil	81.72	11.53	1.53	0.14	0.04	0.57	87.07	1.16
	B	Gravel	83.26	8.92	2.26	0.07	0.21	0.73	77.80	1.10
		Soil	80.89	12.12	1.55	0.14	0.04	0.57	87.50	1.17
	C	Gravel	84.53	8.30	2.17	0.07	0.16	0.66	77.59	1.09
		Rock-Soil	81.11	11.32	2.48	0.11	0.06	0.61	81.11	1.07
	D	Rock	84.17	8.45	3.32	0.09	0.67	0.61	67.17	0.85
	E	Rock	78.15	6.40	1.64	0.07	3.27	0.68	56.21	1.34

Notes: A: surface soil layer; B: strongly weathered layer; C: moderately weathered layer; D: weakly weathered layer; E: bedrock layer; CIA: Chemical Index of Alteration; and Rc: Residual Index.

Table S5-2 Trace Element Content in Black Shale Weathering Profiles (mg kg^{-1})

Profile	Layer	Sample type	S	Cr	Ni	Cu	Zn	Pb	As	Hg	Cd	Se	TC (×10⁻²)
CS003	A	Soil	208	85.8	34.3	134.3	24.1	34.3	30.0	0.41	0.12	3.070	1.53
		Gravel	201	51.2	117.9	206.5	112.7	19.4	100.0	0.22	0.31	2.537	0.82
	B	Soil	178	82.7	36.5	138.5	25.8	33.0	32.2	0.44	0.11	2.757	1.27
		Gravel	143	42.0	54.5	202.9	46.2	15.8	54.6	0.20	0.15	2.328	0.81
	C	Rock	163	28.4	46.6	177.3	26.3	22.9	43.1	0.37	0.16	3.494	0.36
	D	Rock	1434	12.9	13.6	10.2	11.1	12.0	4.1	0.13	0.09	2.245	6.12
	E	Rock	3097	38.9	47.2	34.5	54.9	7.7	5.5	0.08	0.12	0.965	3.39
CS009	A	Soil	170	70.3	24.0	31.0	52.4	17.7	21.6	0.06	0.08	0.534	1.04
	B	Soil	181	81.2	29.7	31.3	64.5	17.8	29.0	0.07	0.09	0.628	0.59
	C	Soil-Rock	236	76.1	38.6	44.9	72.4	18.7	29.0	0.06	0.13	0.783	0.36
	D	Rock	1196	57.4	46.4	75.2	72.8	20.2	23.3	0.05	0.26	1.158	0.92
	E	Rock	3175	69.2	69.5	54.7	96.0	30.1	24.7	0.10	0.68	1.872	1.86
CS010	A	Soil	170	71.5	31.8	38.3	45.9	24.7	36.3	0.11	0.13	1.037	3.26
	B	Soil	251	73.2	39.6	43.1	47.7	23.0	43.1	0.10	0.12	0.871	1.53
		Gravel	100	43.4	23.9	33.3	42.5	15.7	27.9	0.05	0.15	0.632	0.14
	C	Soil-Rock	829	68.9	42.0	48.3	53.5	32.4	34.3	0.12	0.16	1.879	0.58
		Gravel	108	40.4	46.8	39.6	67.2	20.2	35.0	0.05	0.17	0.707	0.14
	D	Rock	5667	67.0	23.2	41.5	17.3	58.5	19.6	0.25	0.13	8.920	0.93
	E	Rock	45400	276.0	105.0	120.2	74.7	206.7	71.2	0.93	0.49	42.402	13.55

Table S6 Trace Element Content Variation Rates (%) at the Rock–Soil Interface of Layer C in Weathering Profiles of Western Zhejiang Black Shales

Profile	S	Cr	Ni	Cu	Zn	Pb	As	Hg	Cd	Se	TC
CS003	-9.6	-191.6	21.6	21.9	1.7	-44.1	25.2	-18.1	26.6	21.1	-250.8
CS009	53.0	-21.5	37.3	26.3	15.4	1.1	-8.6	-125.0	51.0	35.1	-126.1
CS010	81.6	-16.8	-2.5	10.1	8.1	47.0	-71.1	40.7	44.0	73.2	-211.6

Note: The soil layer in the CS003 profile is thin; Layer C soil data was not obtained, and Layer B soil data was used for calculation.

Table S7 Relative Content (%) of Seven-Step Speciation of Cr, Cd, and As in Soil Layers of CS010 Black Shale Profile

Speciation	Cr			Cd			As		
	A	B	C	A	B	C	A	B	C
Water-soluble	0.02%	0.01%	<0.01%	1.45%	1.86%	1.37%	0.20%	0.02%	0.05%
Exchangeable	0.21%	0.36%	0.25%	33.76%	25.73%	31.04%	0.02%	0.02%	0.08%
Carbonate-bound	1.76%	2.10%	1.59%	7.23%	3.45%	0.55%	0.11%	0.14%	0.31%
Humic acid-bound	1.85%	2.10%	1.67%	20.74%	33.95%	50.00%	8.71%	7.52%	7.82%
Fe-Mn oxide-bound	2.56%	2.97%	2.34%	6.11%	2.39%	1.65%	2.83%	3.32%	3.86%
Strong organic-bound	3.25%	2.90%	2.03%	3.22%	2.12%	2.20%	0.15%	0.11%	0.08%
Residual	90.35%	89.57%	92.12%	27.49%	30.50%	13.19%	87.96%	88.88%	87.80%

Table S8 Acid Radical Ions and Heavy Metal Content Characteristics of Surface Water in Changshan Black Shale Area

Water Type	pH	HCO3-	SO42-	As	Al	Ca	Cd	Cl-	Hg	Cr
Rainwater	5.91	6.0	<1.0	<0.004	0.018	0.49	<0.001	<2.0	<0.001	<0.005
Mountain Stream	7.93	102	3.4	<0.004	0.051	19.5	<0.001	<2.0	<0.001	<0.005
Mine Wastewater	3.87	3	309	<0.004	8.2	69.5	0.092	<2.0	<0.001	0.006
Upstream River	7.925	79.5	17.3	<0.004	0.043	24.9	<0.001	10.6	<0.001	<0.005
Downstream River	7.64	76.5	18.8	<0.004	0.13	25.25	<0.001	9.2	<0.001	<0.005
Rainwater	5.5~8.5	-	-	0.05	-	-	0.005	250	0.001	0.1

Water Type	Cu	F-	Fe	K	Mg	Na	Ni	Pb	Se	Zn
Rainwater	<0.005	<0.05	0.02	1.47	0.20	0.16	<0.007	<0.001	<0.004	<0.01
Mountain Stream	<0.005	0.09	0.18	1.60	9.10	0.64	<0.007	<0.001	<0.004	<0.01
Mine Wastewater	0.261	1.34	3.45	2.255	15.63	0.625	0.665	0.002	0.011	2.225
Upstream River	<0.005	0.395	0.075	1.965	4.095	6.875	<0.007	<0.001	<0.004	<0.01
Downstream River	<0.005	0.28	0.16	3.055	3.91	5.975	<0.007	<0.001	<0.004	0.015
Rainwater	1.0	3.00	-	-	-	-	-	0.10	0.02	2.0

Note: pH is dimensionless; other units are in $mg \cdot L^{-1}$.

References

Arthur F.W., Susan L.B. (1995): Chemical weathering rates of silicate minerals. Berlin, Boston, De Gruyter.

Brantley S., Lebedeva M.I. (2011): Learning to read the chemistry of regolith to understand the critical zone. Annual Review of Earth and Planetary Sciences, 39: 387-416.

Brantley S.L., Goldhaber M.B., Ragnarsdottir K.V. (2007): Crossing disciplines and scales to understand the critical zone. Elements, 3: 307-314.

Bufe A., Hovius N., Emberson R., Rugenstein J.K.C., Galy A., Hassenruck-Gudipati H.J., Chang J.-M. (2021): Co-variation of silicate, carbonate and sulfide weathering drives co2 release with erosion. Nature Geoscience, 14: 211-216.

Dixon J.L., von Blanckenburg F. (2012): Soils as pacemakers and limiters of global silicate weathering[j]. Comptes Rendus Geoscience, 344: 597-609.

Jobbágy E.G., Jackson R.B. (2004): The uplift of soil nutrients by plants: Biogeochemical consequences across scales. 85: 2380-2389.

Kalnicky D.J., Singhvi R. (2001): Field portable xrf analysis of environmental samples. J Hazard Mater, 83: 93-122.

Li C., HAN L., YANG J., GAO J., Lv B. (2024): Distribution and constraints of rare earth elements in granite weathering profile from the subtropical coastal region of southeast china %j earth and environment.

Lijun L. (2012): Genetie characteristies and taxonomic classification of hilly soils in the western zhejiang province. Zhejiang University.

Lin S., Wu X., Sun C., Miao X. (2017): Research on micro-mechanism and evolution of black shale in chemical weathering process. Journal of Chengdu University of Technology: Science & Technology Edition, 44: 10.

Mao J., Xie G., Li X., Zhang C., Mei Y. (2004): Mesozoic large scale mineralization and multiple lithospheric extension in south china[j]. Earth Science Frontiers, 11: 45-55.

Mao J., Li X., Li H., Qu X., Zhang C., Xue C., Wang Z., Yu J., Zhang Z., Feng C. (2005): Types and characteristics of endogenetic metallic deposits in orogenic belts in china and their metallogenic processes[j]. Acta Geologica Sinica, 79: 342-372.

Nesbitt H.W., Young G.M. (1984): Prediction of some weathering trends of plutonic and volcanic rocks based on thermodynamic and kinetic considerations. Geochimica et Cosmochimica Acta, 48: 1523-1534.

Shukui L., Yongmei D. (2015): Determination of heavy metal elements in soil by icp-ms[j]. Chinese Journal of Inorganic Analytical Chemistry, 5: 4.

Wang M. (2022): Geochemical characteristics and influencing factors of selenium-enriched soilsin cultivated land around typical stone coal mines in western zhejiang[j]. Geoscience: 036.

Wang Q., Dong Y., Zheng W., Zhou G. (2007): Soll geochemical baseline values and environmental background val- ues in zhejiang, china[j]. 26: 590-597.

Wei W., Ling S., Li X., Sun C., Feng J., Luo J., Wu X., He C. (2025): Mineral-dependent release, migration and enrichment of toxic elements during black shale weathering: An

integrated study from profile scale to mineral scale. Journal of Hazardous Materials, 487: 137119.

Wu Y., Tian H., Gong D., Li T., Zhou Q. (2020): Paleo-environmental variation and its control on organic matter enrichment of black shales from shallow shelf to slope regions on the upper yangtze platform during cambrian stage 3. Palaeogeography, Palaeoclimatology, Palaeoecology, 545: 109653.
